# RME-1-associated recycling endosomes participate in vitellogenin secretion in *Caenorhabditis elegans*

**DOI:** 10.1093/lifemeta/loaf026

**Published:** 2025-06-28

**Authors:** Chao Zhai, Meng-Qiu Dong

**Affiliations:** National Institute of Biological Sciences, Beijing 102206, China; Tsinghua Institute of Multidisciplinary Biomedical Research, Tsinghua University, Beijing 100084, China; National Institute of Biological Sciences, Beijing 102206, China; Tsinghua Institute of Multidisciplinary Biomedical Research, Tsinghua University, Beijing 100084, China

**Keywords:** vitellogenin, yolk protein, secretion, recycling endosomes, *Caenorhabditis elegans*, apoB-100

## Abstract

Vitellogenins (VITs), the lipoprotein precursors of yolk proteins in *Caenorhabditis elegans*, are expressed in the intestine, secreted into the pseudocoelom, and ultimately transported into oocytes. However, the mechanism by which VITs are secreted out of the intestine remains unclear. In this study, a candidate RNA interference (RNAi) screen suggested that both the conventional secretion pathway and recycling endosomes (REs) are essential for VIT secretion. In addition to expected conventional secretion, VITs were also found to be synthesized in the intestinal rough endoplasmic reticulum (ER) and then transported to the Golgi apparatus. VIT-2::GFP accumulated in enlarged REs upon depletion of receptor-mediated endocytosis 1 (RME-1), a key protein that facilitates endocytic recycling. Moreover, the number of VIT-2::GFP-containing REs decreased upon inhibition of either ER-to-Golgi trafficking, trans-Golgi-to-endosome trafficking, or endocytosis. These findings suggested that REs are required for intestinal secretion of both newly synthesized VITs and yolk proteins endocytosed from the pseudocoelom. Moreover, RME-1 was found at the periphery of vitellogenin-containing vesicles (VVs), and this required RAB-10, the upstream regulator of RME-1 in endocytic recycling. RAB-10 was additionally required for the trafficking of VV from the apical/luminal side to the basal/pseudocoelomic side of the intestine. Together, these results identify REs as an intermediate compartment for the secretion of VIT/yolk proteins out of the intestine, suggesting a conserved role for endocytic recycling in the secretion of lipoproteins in mammals, particularly those assembled by apolipoprotein B-100 (apoB-100), a mammalian homolog of VITs.

## Introduction

Vitellogenins (VITs) are lipoprotein precursors of yolk proteins that are widely expressed in oviparous animals [1–3]. VITs are synthesized in the soma and transported to the reproductive system via fluid circulation, where they deposit lipids in the growing oocytes for provisioning progeny [1, 2]. In addition to nourishing functions, VITs also play diverse and critical roles in caste establishment in social animals, antioxidant stress, defense against pathogen infection, and regulation of lifespan [4–10].

In humans, the large lipid transfer protein, apolipoprotein B-100 (apoB-100), is a VIT homolog [11]. It is synthesized in the liver and provides the basic scaffolding for lipid transport via circulation to other tissues as low-density lipoproteins (LDL) or very-low-density lipoproteins (VLDL) [12]. High blood LDL or VLDL levels are widely accepted to initiate and aggravate atherosclerosis, leading to cardiovascular diseases, a major cause of mortality worldwide [13–15]. A mechanistic understanding of lipoprotein secretion can thus enable the development of both preventative and therapeutic treatments for atherosclerosis.

The *Caenorhabditis elegans* genome contains six *vit* genes (i.e. *vit-1* to *vit-6*), and their protein products are the most abundantly expressed ones in *C. elegans* hermaphrodites [1, 9, 16]. As lipoproteins, VITs are synthesized in the nematode intestine and secreted into the pseudocoelom, where they are subsequently cleaved to form mature yolk proteins, and finally delivered to oocytes via RME-2 (receptor-mediated endocytosis 2)-mediated endocytosis [1, 17, 18]. Although VIT synthesis in the intestine was demonstrated longer than four decades ago [17], it remains unclear how VITs are secreted from the intestine, an essential process in resource reallocation between maternal soma and progeny in *C. elegans,* which may also shed light on lipid-binding protein secretion in other species, especially apolipoproteins in humans.

Previous nematode studies have shown that knockdown of endoplasmic reticulum exit site (ERES) proteins, for example, SFT-4, SEC-13, SEC-23, and TNGL-1, results in VIT accumulation in the intestinal endoplasmic reticulum (ER) lumen, suggesting that VITs are synthesized in the intestinal rough ER [19–21]. Although a genome-wide RNA interference (RNAi) screening identified 100 candidate proteins potentially involved in VIT secretion, only a small portion actually contributed to protein secretion, including ERES assembly and ER−Golgi antegrade/retrograde transport genes [20]. Consistent with these findings, immuno-electron microscopy (immuno-EM) using anti-VIT antibodies revealed that VITs in the *C. elegans* intestine always organize into vesicle structures and localize along the classic exocytosis route, encompassing the rough ER, Golgi, single membrane-bounded vitellogenin-containing vesicles (VVs), and pit structures on the basal membrane of the intestine [22]. Working models of conventional secretion propose that secretory proteins are synthesized in the rough ER, and then transported to the Golgi for processing and encapsulation in trans-Golgi network (TGN)-derived vesicles, which ultimately tether to and fuse with the plasma membrane (PM) for secretion [23]. The evidence collectively suggests that the conventional secretion pathway mediates VIT secretion in *C. elegans*.

Recycling endosomes (REs) are produced through the maturation of early endosomes and mediate the recycling of receptors back to the PM for subsequent rounds of endocytosis [24]. Intriguingly, REs can reportedly serve as an intermediate compartment for certain Golgi-derived secretory proteins prior to secretion in cultured mammalian cells [25, 26]. Earlier studies showed that the *C. elegans* intestine could endocytose pseudocoelomic molecules, such as an exogenously administered fluorescent dye FM4-64 or a green fluorescent protein tagged with a signal peptide sequence that was originally expressed in body wall muscles and then released to the pseudocoelom [27]. RAB-10, a small GTPase, and its effector protein EHBP-1 (a calponin homology domain protein) are shown in a recent study to facilitate exocytic trafficking of VIT-2 by tethering post-Golgi vesicles to REs prior to secretion [28]. In other words, REs are an important depot for VIT secretion, which, as Liu *et al.* demonstrate, involves more than the conventional secretion pathway [28].

**Figure 1:**
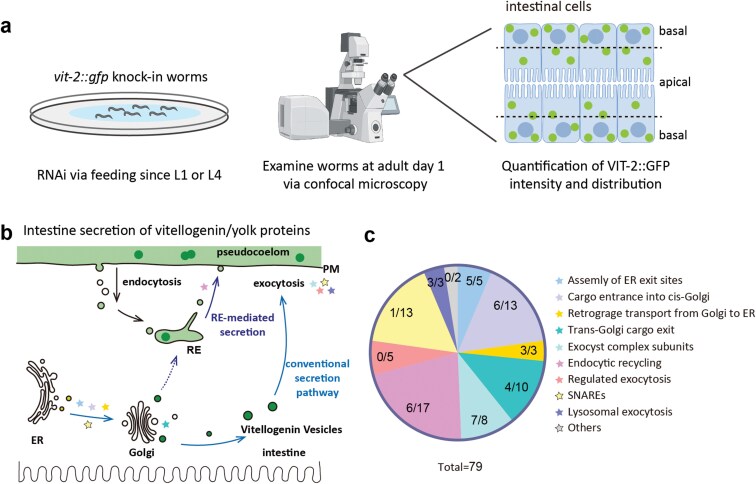
Candidate RNAi screening to identify pathways regulating VIT secretion in *C. elegans*. (a) Workflow of RNAi screen. (b and c) Pathways of vesicle trafficking in which the candidate genes are involved, marked with different-colored pentagrams (b), corresponding to functional classification of candidate genes (c). Each segment shows the ratio of positive readouts (numerator) to total candidates (denominator) for a given function. SNAREs: soluble N-ethylmaleimide-sensitive factor attachment protein receptors.

To explore the mechanism(s) of VIT secretion in *C. elegans*, in the present study, we selected 79 genes for individual knockdown by RNAi in worms expressing VIT-2::GFP at the endogenous *vit-2* locus. These genes were previously identified to participate in vesicle trafficking. VIT accumulation in the intestine was quantified by the mean signal intensity of VIT-2::GFP, and the distribution of VITs within the intestine was assessed by VIT-2::GFP intensity at the apical versus the total of the two sides (Fig. 1a; Supplementary Fig. S1). This screen confirmed that the conventional secretion pathway and REs are both required for VIT secretion. Additionally, we verified that VITs are synthesized in the intestinal rough ER and delivered to the Golgi. Consistent with previous findings [27, 28], our data indicate that VITs from the TGN can be transported to REs in addition to the PM, and pseudocoelomic yolk can be endocytosed by the intestine and secreted back out via intestinal REs. This study may provide a theoretical basis for investigating the secretion of lipid transfer proteins, especially apoB-100, in mammals.

## Results

### RNAi screen identifies both the conventional secretion pathway and REs in VIT-2::GFP secretion

To initiate a screen of genes required for VIT secretion in *C. elegans*, we selected 79 candidate genes previously identified to participate in vesicle trafficking (see Supplementary Table S1 for an overview). These candidate genes were subsequently classified into ten groups based on their annotated functions, such as assembly of ERES, cargo entrance into *cis*-Golgi, and trans-Golgi cargo exit (Fig. 1b and c). After feeding adult worms expressing a VIT-2::GFP reporter with siRNAs targeting each respective candidate, we used confocal microscopy to quantify the VIT-2::GFP signal in the intestine as a means of assessing potential alterations or defects in VIT-2 secretion (see Materials and Methods section “Quantification of intestinal VIT-2::GFP fluorescent intensity” and Supplementary Fig. S1). This screen identified 35 genes from among the 79 candidates, which resulted in altered VIT-2::GFP secretion phenotype upon transient knockdown by RNAi (Fig. 1b and c). Based on overlap in their reported functions, we hypothesized that the conventional secretion pathway, together with REs, likely mediates VIT secretion in *C. elegans* (Fig. 1b and c; Supplementary Table S1).

### The conventional secretion pathway mediates VIT secretion

**Figure 2:**
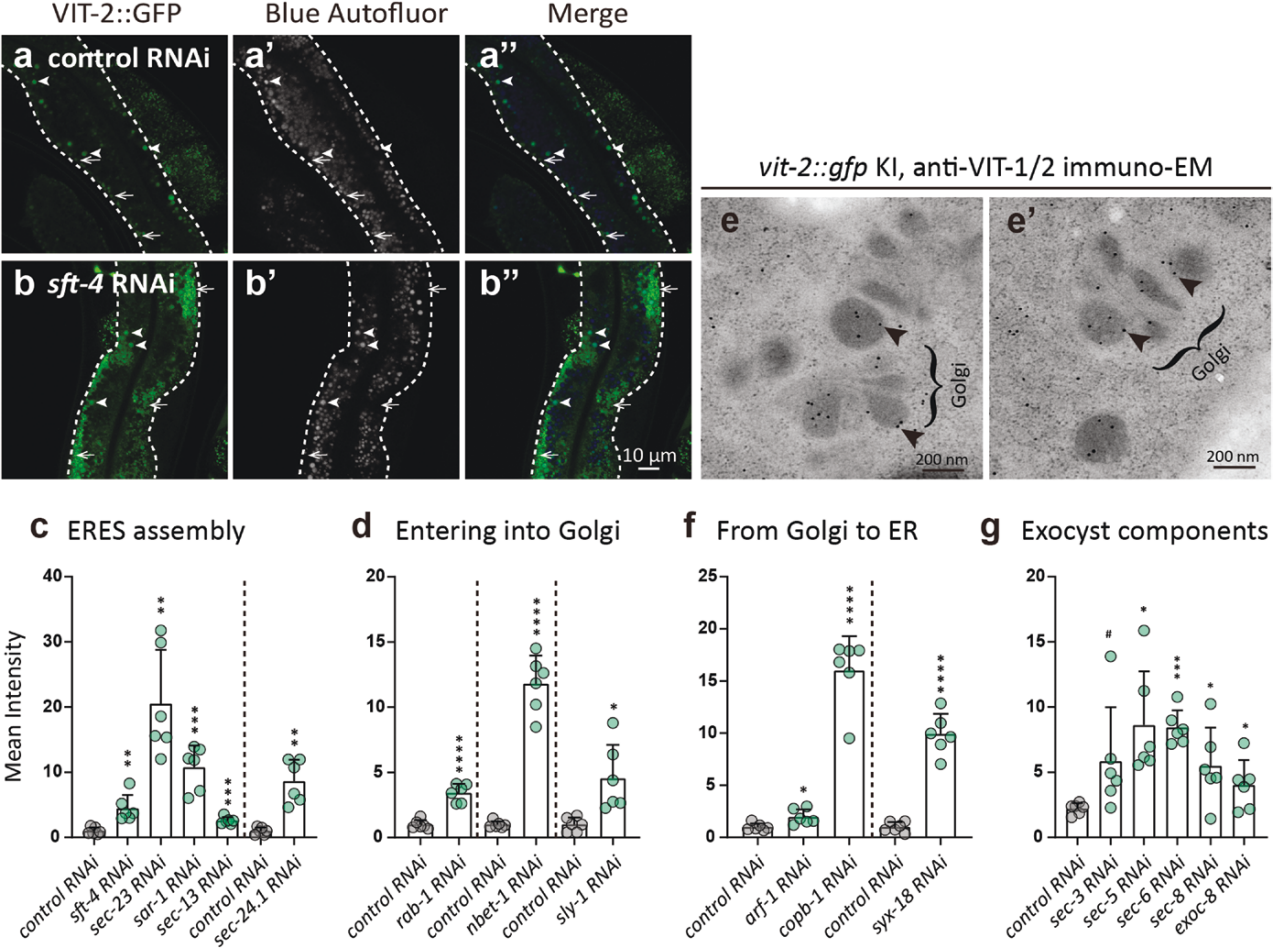
Silencing genes involved in the conventional secretion pathway leads to VIT-2::GFP accumulation in the intestine. (a–b’’) Confocal images of intestinal VIT-2::GFP in control (a–a’’) and *sft-4* RNAi (b–b’’) worms at AD 1 (RNAi initiated at early L4). Autofluor means autofluorescence from gut granules (white arrowheads). White arrows point to VIT-2::GFP⁺ VVs. KI means knock-in. (c, d, f, and g) Bar plots quantifying mean VIT-2::GFP intensity, with dashed lines separating trials. Each data point represents one value from one worm image. Six images of six worms were analyzed for each bar. Statistical significance: ^#^*P* < 0.1; ^*^P < 0.05; ^**^P < 0.01; ^***^P < 0.001; ^****^P < 0.0001 by *t*-test. (e and e’) Anti-VIT-1/2 immuno-EM images showing 10-nm gold particles (arrowheads) in Golgi cisternae (braces).

**Figure 3:**
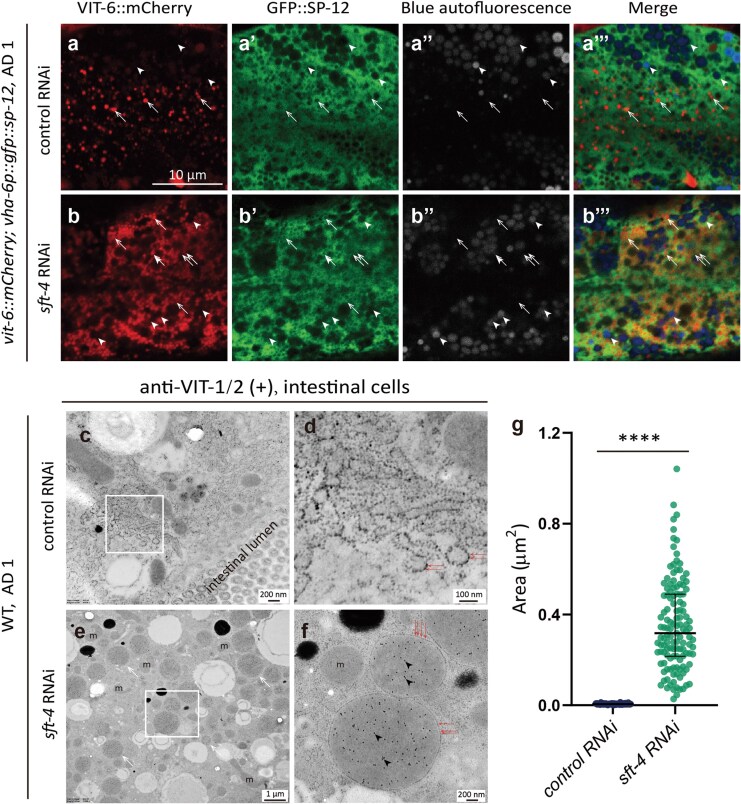
SFT-4 depletion leads to VIT retention in the intestinal rough ER lumen. (a–b’’’) *sft-4* RNAi increases intestinal VIT-6::mCherry signal and its colocalization with ER marker GFP::SP-12 (control: empty vector L4440). White arrows: VIT-containing structures; white arrowheads: gut granules. (c–f) Ribosomes on ER membrane are shown by red arrows. Images in (d) and (f) show magnified views of boxed regions in (c) and (e), respectively. The dot plot quantifies ER lumen areas; each dot represents one ER membrane-enclosed region, and nearly all ER membrane-enclosed regions in one image were measured (five images per group). Statistical significance: ^****^P < 0.0001 by *t*-test.

Given our above results, we then focused on validating the functions of these candidates in VIT secretion. In *C. elegans*, the conventional secretion pathway begins with protein synthesis in the rough ER. Freshly synthesized proteins are packaged into COPⅡ (coat protein complex Ⅱ) vesicles and exported from the ER via the ERES. VITs reportedly accumulate inside the ER lumen following *sft-4*, *sec-23*, or *sar-1* silencing [19]. Additionally, TNGL-1—the *C. elegans* ortholog of TANGO1 (transport and Golgi organization protein 1)—was recently demonstrated to play an essential role in VIT export from the ER [21]. In the present study, knockdown of known ERES assembly genes (e.g., *sft-4*, *sec-13*, *sec-23*, *sec-24.1*, and *sar-1*) resulted in a significant increase in VIT-2::GFP signal intensity in the intestine (Fig. 2a–c; Supplementary Fig. S2a–f’’). VIT-6::mCherry also accumulated in the intestinal ER lumen of *sft-4* RNAi worms (Fig. 3a–b’’’). Examination of *sft-4* knockdown worms by immuno-electron microscopy (immuno-EM) imaging revealed luminally enlarged rough ER, labeled with the anti-VIT-1/2 gold particles and decorated with ribosomes on its membrane, whereas control worms treated with untargeted siRNAs displayed no such enlargement of rough ER lumen (Fig. 3c–g). These results suggest that VITs are synthesized in the rough ER of intestinal cells, and their export requires ERES proteins.

After ER synthesis, cargo proteins of the conventional secretion pathway are transported into the Golgi via COPⅡ vesicles, which fuse with the ER–Golgi intermediate compartment (ERGIC) or *cis*-Golgi membranes in a manner dependent on membrane-localized small GTPase RAB-1 and Golgins [29, 30]. The secretory proteins subsequently traverse the Golgi as the *cis*-cisternae mature into *trans*-cisternae [31]. To replenish *cis*-cisternae, retrograde transport recycles resident proteins from *trans*-Golgi to *cis*-Golgi and potentially all the way back to the ER [31, 32]. We observed that knockdown of these candidate genes required for entrance to the Golgi or the retrograde transport from the Golgi to the ER resulted in VIT-2::GFP retention in the intestine (Fig. 2d and f; Supplementary Fig. S2g–q’’). Moreover, anti-VIT-1/2 immuno-gold particles could be detected in the immuno-EM images of the intestinal Golgi cisternae (Fig. 2e and e’), suggesting that VITs are likely transported into the Golgi for processing.

After reaching the TGN, conventional secretory proteins are exported from the Golgi via membrane budding, and ultimately form secretory vesicles. Secretory vesicles are then transported to the PM, where membrane-associated exocyst and the soluble N-ethylmaleimide-sensitive factor attachment protein receptors (SNAREs) facilitate their tethering and fusion with the PM [33, 34]. Confocal microscopy assays indicated that worms with exocyst subunit knockdown (i.e. *sec-3, sec-5, sec-6, sec-8*, or *exoc-8*) displayed increased VIT-2::GFP signal in the intestine to varying extents (Fig. 2g; Supplementary Fig. S2r–w), suggesting that exocyst-mediated exocytosis could directly or indirectly regulate VIT secretion in *C. elegans*. These cumulative results showed that silencing genes involved in cargo export from the ER, entrance into the Golgi, or exocyst assembly all led to intestinal retention of VIT-2::GFP, strongly supporting our hypothesis that the conventional secretion pathway mediates VIT secretion.

### The basolateral REs in the intestine contribute to VIT secretion

**Figure 4:**
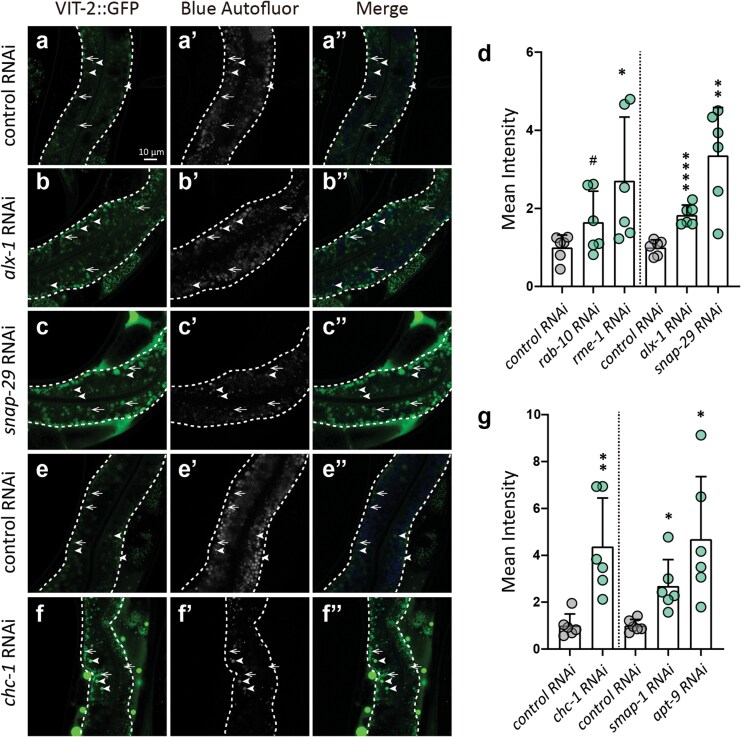
Silencing endocytic recycling genes increases the intestinal VIT-2::GFP intensity. (a–c, e, and f) RNAi initiated at early L4, with imaging conducted at AD 1. Arrows: VIT-2::GFP puncta; arrowheads: gut granules; white dashed lines: intestinal outline. (d and g) Box plots displaying the mean and SD, with dashed lines separating trials. Each point represents one worm measurement. Six images of six worms were analyzed for each bar. Statistical significance: ^#^*P* < 0.1; ^*^P < 0.05; ^**^P < 0.01; ^****^P < 0.0001 by *t*-test.

As TGN-derived cargoes are reportedly transported into REs before reaching the PM in worms and mammalian cells [25, 28], we next investigated whether REs or the entire way of endocytic recycling plays a role in VIT secretion in *C. elegans.* To this end, we induced knockdown of genes known to participate in basolateral endocytic recycling in the intestine, including *rab-10*, a small GTPase that promotes maturation of some early endosomes into REs [35–37]; *rme-1*, an Epsin-15 homology (EH) domain gene that facilitates membrane tubulation of REs and recycling of endocytic cargos back to the PM [27, 38]; *alx-1,* which interacts with RME-1 to mediate endocytic recycling of basolateral cargos in the *C. elegans* intestine [39]; and *snap-29* (synaptosomal-associated protein 29), encoding a SNARE required for post-Golgi vesicle fusion with REs to coordinate cargo delivery to the cell surface in *C. elegans* [40, 41]. Knockdown of each respective gene caused intestinal VIT-2::GFP accumulation (Fig. 4a–d). On the contrary, knockdown of other genes involved in early endosome maturation to REs (e.g. *arf-6*, *sac-1*, *ptrn-1*, and *tbc-2*) [36, 42–44], cargo sorting within REs (e.g. *sdpn-1*) [45, 46], tubulation or vesiculation of RE membrane (e.g. *rtkn-1*, *sid-3*, and *amph-1*) [47–49] had no effects on the VIT-2::GFP secretion in the intestine (Supplementary Table S1). Taken together, these results suggest that REs are involved in VIT secretion.

The TGN and endosomes are dynamic sorting compartments that communicate with each other through vesicle trafficking [50, 51]. Previous reports suggest that clathrin-coated post-Golgi vesicles may be transported towards endosomes, and some of them mature into REs. This process requires clathrin adaptor proteins such as the AP-1 (adaptor protein complex 1) and GGAs (Golgi-localized, Gamma-ear-containing, Arf-binding proteins), which recognize and bind cytosolic sorting motifs on cargo proteins processed in the TGN [52]. In *C. elegans*, the stromal membrane-associated protein-1 (SMAP-1) is reportedly essential for clathrin assembly at the TGN and facilitates APT-9 localization at the TGN [53]. APT-9 is the only *C. elegans* ortholog of human GGA1 known to mediate TGN-derived vesicle trafficking to endosomes [51, 53]. Knockdown of *chc-1* (clathrin heavy chain), *smap-1*, or *apt-9* could increase VIT-2::GFP signal in the intestine (Fig. 4e–g), which supports the idea that REs contribute to VIT secretion.

### RME-1 depletion induces VIT accumulation within enlarged REs

**Figure 5:**
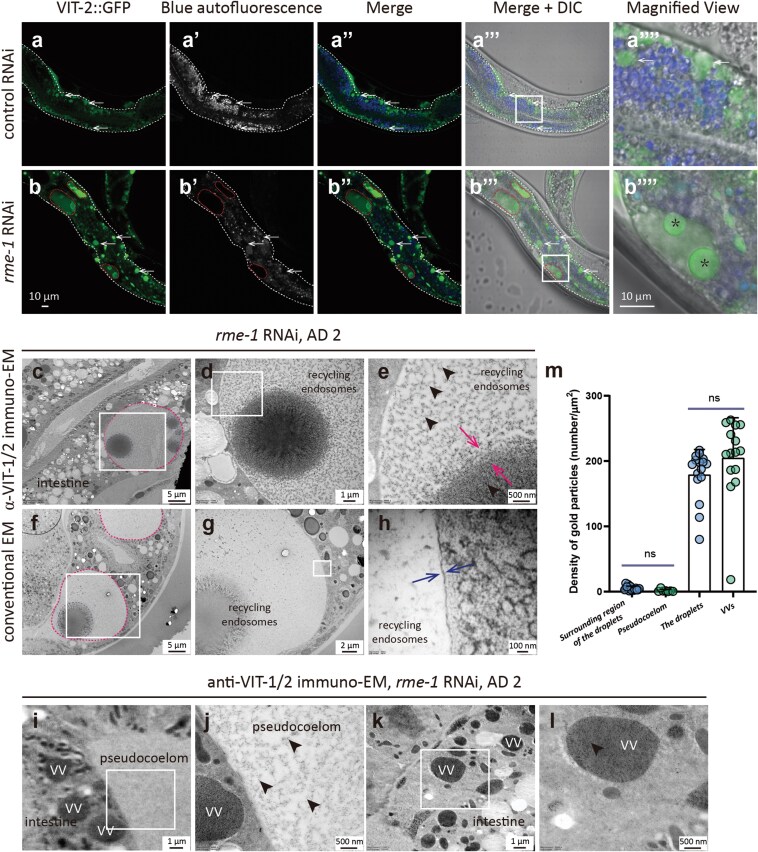
The enlarged REs in the intestine contain VIT-2::GFP droplet structures upon depletion of RME-1. (a and b) VVs indicated by white arrows. Images in (a’’’’) and (b’’’’) are the magnified views of the white rectangular region in (a’’’) and (b’’’), respectively. (b–l) Red dashed lines profile the enlarged REs, these vacuole-like structures in (b), of *rme-1* RNAi worms. ^*^Asterisks indicate VIT-2::GFP droplets inside the enlarged REs. The blue dashed lines outline the droplet structures. Black arrowheads indicate the 10-nm gold particles. Paired red arrows point to the boundary between the droplet structures and their surrounding region. Blue-paired arrows point to the lipid-bilayer membrane structures of an enlarged RE. The right images are magnified images of the rectangles in the left images. (m) Mean and SD are shown. Each point means one value calculated based on one image, and 14, 7, 16, and 15 images were used for the four groups, respectively, in (m). ns, no significance by *t*-test analysis.

To further verify the role of REs in VIT secretion, we knocked down *rme-1*, a key regulator of RE formation and function, and examined the effects on intracellular VIT localization. Consistent with previous reports, we found that the intestines of *rme-1* RNAi worms harbored abundant enlarged REs, which appeared as vacuolar structures [27, 28, 39, 49]. Further, almost all of these enlarged REs contained both diffused VIT-2::GFP as well as VIT-2::GFP droplets (Fig. 5a–b’’’’; Supplementary Fig. S3a and a’), the latter of which could be observed to aggregate within REs (Supplementary Video 1). Moreover, we could also observe the formation of similar VIT-6::mCherry droplets in worms expressing that alternative reporter (Supplementary Fig. S3b–e). We noted that these VIT-2::GFP droplets in the REs resembled VVs (Fig. 5a–a’’’’), which were described in our previous work [22, 54].

Using conventional EM and immuno-EM methods, we then characterized the enlarged REs in *rme-1* RNAi worms. These image data confirmed the membrane enclosure of the enlarged structures and revealed that the droplet structures inside lacked membranes (Fig. 5c–h). Consistent with the GFP signal in our above confocal microscopy assays, the droplets and their surrounding region could be labeled with anti-VIT-1/2 gold particles, and the labeling density of droplets was significantly higher compared to gold labeling of the surrounding region (Fig. 5c–e, m). Further analysis indicated that VV labeling density was similarly higher than that of the pseudocoelom in EM images, and this difference in signal was comparable to that of VIT-2::GFP droplets in REs (Fig. 5c–e, i–m), implying that these droplets might represent the contents of VVs that had been delivered to the enlarged REs.

### Endocytosed pseudocoelomic yolk is secreted via RME-1-associated REs

**Figure 6:**
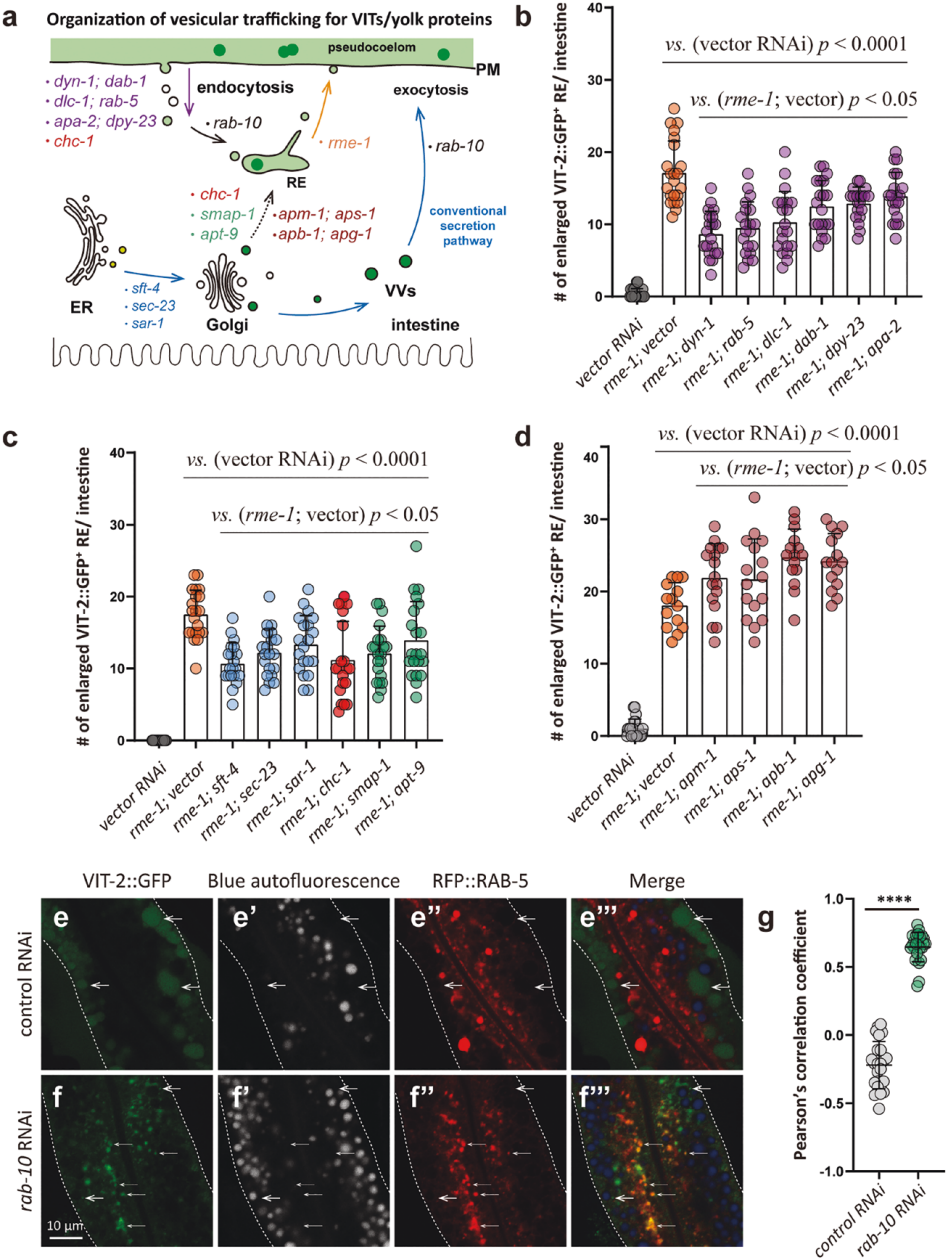
Endocytosed yolk and Golgi-derived VITs can be delivered into the intestinal enlarged REs of *rme-1* RNAi worms. (a) Diagram of vesicle-mediated trafficking, with genes involved in different processes marked by different colors. (b–d) *rme-1* RNAi initiated at L1. Other genes were knocked down from early L4. Box plots show the mean and SD, with each point representing one value of one intestine (15–20 worms per group). (e and f) RNAi treatment started at early L4. Worms were imaged at AD 1. Thick white arrows indicate VIT-2::GFP puncta; thin white arrows show the colocalization between VIT-2::GFP puncta and RFP::RAB-5-labeled endosomes. White dashed lines outline the intestine. (g) The dot plot shows the mean ± SD, with each point representing the one value measured from one ROI, four ROIs per image (six images from six worms per group). ns, no significance; ^****^P < 0.0001 by *t*-test analysis in (b–d and g).

Pseudocoelomic substances undergo endocytosis in the *C. elegans* intestine [27]. Among such substances, yolk proteins are cleaved from VITs upon secretion into the pseudocoelom and are subsequently widely distributed throughout the pseudocoelom [18]. In this context, we speculated that VITs with enlarged REs in *rme-1* RNAi worms might be pseudocoelomic yolk proteins that were endocytosed by the intestine, or alternatively, they may be newly synthesized and exported from the Golgi. To determine whether these RE-associated VITs were derived from the pseudocoelom, we induced knockdown of endocytosis genes, including *dyn-1*, *rab-5*, *dlc-1*, *dab-1*, *dpy-23*, or *apa-2,* conjointly with *rme-1* silencing. In *rme-1* knockdown worms, suppressing any of these respective genes resulted in a significant decrease in the number of enlarged VIT-2::GFP-positive REs (Fig. 6a and b), indicating that VITs in the enlarged REs of *rme-1* RNAi worms originated from the pseudocoelom.

RAB-10 activity in early endosome maturation into REs precedes RME-1 functions in the endocytic recycling pathway [36, 37]. To verify that pseudocoelomic yolk was present in early endosomes, we knocked down *rab-10* in worms expressing *vit-2::gfp; vha-6p::rfp::rab-5* to co-label early endosomes along with VIT-2. Subsequent imaging assays indicated that *rab-10* silencing resulted in significantly increased abundance of VIT-2::GFP puncta colocalized with RFP::RAB-5-labeled endosomes compared to that in non-targeted RNAi controls (Fig. 6e–g), suggesting that the *C. elegans* intestine could take up pseudocoelomic yolk proteins via endocytosis. Taken together, these results indicate that pseudocoelomic yolk proteins endocytosed by the intestine could be secreted via REs in *C. elegans.*

### The Golgi-derived VITs are delivered to REs in the intestine

To determine whether newly synthesized VITs derived from the Golgi are also incorporated into the REs, we knocked down genes involved in ERES assembly or TGN sorting to endosomes in *rme-1* RNAi worms. We found that inhibiting secretory cargos from exiting the ER led to a decrease in the number of enlarged VIT-2::GFP-positive REs that formed in the absence of RME-1 (Fig. 6c; Supplementary Fig. S3f).

As mentioned above, GGAs and AP-1 promote clathrin recruitment and formation of clathrin-coated vesicles at trans-Golgi that are believed to be transported to endosomes in mammalian cells [51, 52, 55], whereas the *C. elegans* homologs of GGAs, AP-1 complex, and clathrin localize to the TGN to participate in protein secretion and polarization of intestinal epithelium [53, 56, 57]. SMAP-1 also facilitates TGN sorting via APT-9 and clathrin recruitment to the TGN [53]. Inhibiting genes such as *chc-1*, *smap-1*, and *apt-9,* which potentially function in TGN sorting to endosomes in *C. elegans*, resulted in a decrease in the number of REs (Fig. 6c; Supplementary Fig. S3f and g). However, silencing AP-1 subunits increased RE abundance (Fig. 6d; Supplementary Fig. S3g), probably because *C. elegans* AP-1 complexes have different functions in vesicle trafficking as they do in mammals.

It is worth noting that knockdown of the clathrin heavy chain gene, *chc-1*, decreased the number of enlarged VIT-2::GFP-positive REs (Fig. 6c), potentially due to inhibition of endocytosis and TGN trafficking to endosomes, as CHC-1 has been shown to participate in both clathrin coating of early endosomes and clathrin coating of TGN-derived vesicles [58]. Collectively, these observations suggested that Golgi-derived VITs are also delivered to REs in the *C. elegans* intestine.

### RME-1 puncta localize at the VV periphery

**Figure 7:**
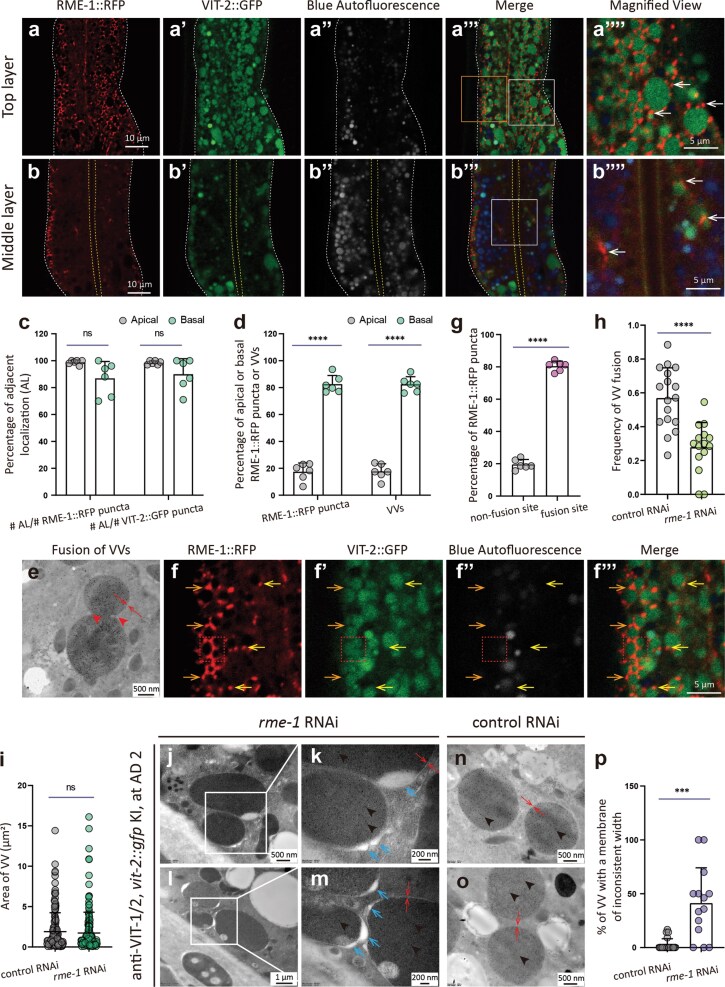
RME-1::RFP puncta localize at VV periphery and may facilitate membrane recycling during fusion. (a–b’’’’) *vit-2::gfp; vha-6p::rme-1::rfp* worms at AD 1 show RME-1::RFP distribution (white arrows), with basal/apical membranes marked by white/yellow dashed lines. (e) An immuno-EM image capturing two VVs undergoing fusion. Red arrows indicate the lipid-bilayer membrane, which is one continuous membrane at the fusion site. (f–f’’’) Probable VV fusion events as captured by epi-fluorescent microscopy. A VIT-2::GFP-containing vesicle not engaging in fusion is characteristically round in shape; those in the process of fusion are not, as shown in the rectangular region outlined by dotted red lines, in which the one on the left touches the two on the right. There is no discernible gap between the one on the left and either of the two on the right. There are multiple probable VV fusion events in the image. (f–f’’’) Magnified views of the orange rectangle in (a’’’). RME-1::RFP at fusion (orange arrows) versus non-fusion sites (yellow arrows) are shown. (c, d, and g) Quantification from random regions (approximately 400 μm²; six images from six worms). (h, i, and p) Immuno-EM analysis of basal VVs (h) or all VVs (i and p) in one image (control: 20–21 images; *rme-1* RNAi: 14–15 images). (j–o) VV membranes with inconsistent width (blue arrows) and gold particles (arrowheads). *rme-1* RNAi was conducted since L1. ns, no significance; ^***^P < 0.001; ^****^P < 0.0001 by *t*-test.

We then sought to determine whether VITs are localized in REs of worms cultured under standard conditions. Towards this objective, we examined VIT-2::GFP and RME-1::RFP localization in worms expressing *vit-2::gfp; vha-6p::rme-1::rfp.* Confocal imaging data showed that RME-1::RFP puncta localized at the periphery of VIT-2::GFP puncta (Fig. 7a–c), which were previously identified as VVs using anti-VIT-1/2 immuno-EM [22, 54]. We also noted that nearly every VV had at least one adjacently localized RME-1::mCherry punctum, *vice versa* (Fig. 7c), and these RME-1-associated VVs were present in significantly greater numbers on the basal side compared to the apical side (Fig. 7a–b’’’’, d), collectively suggesting that VVs were closely associated with REs [27, 39, 46, 49, 59].

We then investigated the function of these VV-associated RME-1 puncta. Consistent with our previous findings [54], we observed that VVs could fuse with each other, and the examples of VV fusion are shown in Figure 7e–f’’’’. Additionally, RME-1::RFP puncta exhibited preferential localization (approximately 80%) at the fusion sites between VVs compared to those detected at non-fusion sites on the VV exterior (approximately 20%) (Fig. 7f and g). To investigate whether RME-1 plays a role in VV fusion, we quantified the fusion events along the basal membrane of the intestine in our immuno-EM and fluorescent images. This analysis revealed that *rme-1* RNAi worms had fewer VV fusion events than corresponding regions of non-targeted RNAi control worms (Fig. 7h), although the size of VVs did not significantly differ between the knockdown and control animals (Fig. 7i; Supplementary Fig. S4a). At the same time, we observed that the VV membranes displayed inconsistent width at different sites in *rme-1* knockdown worms, but not in the VVs of controls (Fig. 7j–p). These results suggest that RME-1 might promote the sloughing of excess VV membrane during fusion with other VVs, which is consistent with the known function of RME-1 on REs [38].

### RAB-10 facilitates VV trafficking from the apical side to the basal side of the intestine

**Figure 8:**
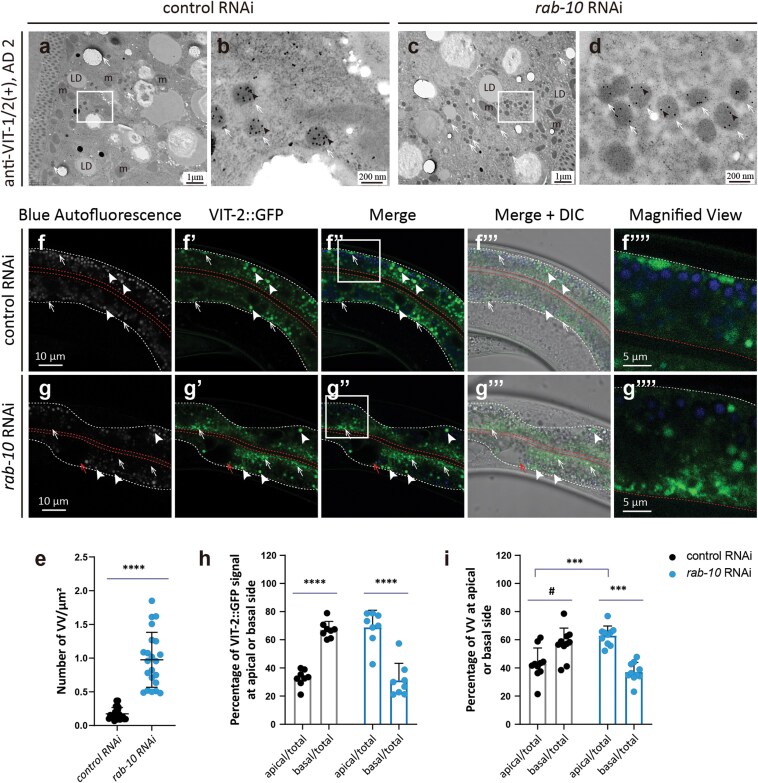
RAB-10 is required for VV trafficking from the apical side to the basal side of the intestine. (a–d) VVs in WT *C. elegans* at AD 2 are indicated by white arrows. Black arrowheads mark 10-nm gold particles. Images in (b) and (d) are magnified views of the rectangles in (a) and (c), respectively. (f–g’’’’) The basal and apical intestinal membranes are marked by white and red dashed lines, respectively. White arrows indicate VIT-2::GFP puncta or VVs; white arrowheads mark gut granules with blue and green autofluorescence. The red arrow points to an abnormal endosome. Images in (f’’’’) and (g’’’’) are magnified views of the rectangles in (f’’) and (g’’), respectively. (e, h, and i) Mean and SD. Each point represents a value from one immuno-EM image (e and i) or one fluorescent image (h). Specifically, 22, 8, and 10 images were analyzed for each group in (e), (h), and (i), respectively. ^#^*P* < 0.1; ^***^P < 0.001; ^****^P < 0.0001 by *t*-test analysis.

As the above screen showed that knockdown of the small GTPase, *rab-10*, which functions as an upstream regulator of RME-1 in endocytic recycling in *C. elegans* [34], led to a slight increase in intestinal VIT-2::GFP signal (Fig. 4d), we further examined VVs in *rab-10* RNAi worms. Consistent with our screen and previous EM data [28], immuno-EM imaging showed that *rab-10* knockdown worms accumulated significantly more VVs in the intestine compared to non-targeted RNAi controls (Fig. 8a–e; Supplementary Fig. S4b and c). Furthermore, RAB-10 depletion resulted in greater distribution of VIT-2::GFP signals on the apical side of the intestine, whereas VIT-2::GFP predominantly localized at the basal side in controls (Fig. 8f–h). Employing anti-VIT-1/2 immuno-EM, we found that VVs exhibited the same patterns of asymmetric distribution between knockdown and control animals (Fig. 8i; Supplementary Fig. S4b and c). These results suggested that RAB-10 may facilitate VV trafficking from the apical side to the basal side of the intestine prior to secretion, resembling the function of Rab10 in *Drosophila,* which cooperates with kinesins to mediate vesicle trafficking along microtubules to the basal surface of follicular epithelial cells [60].

### RME-1 puncta are localized at the VV periphery in a RAB-10 dependent manner

**Figure 9:**
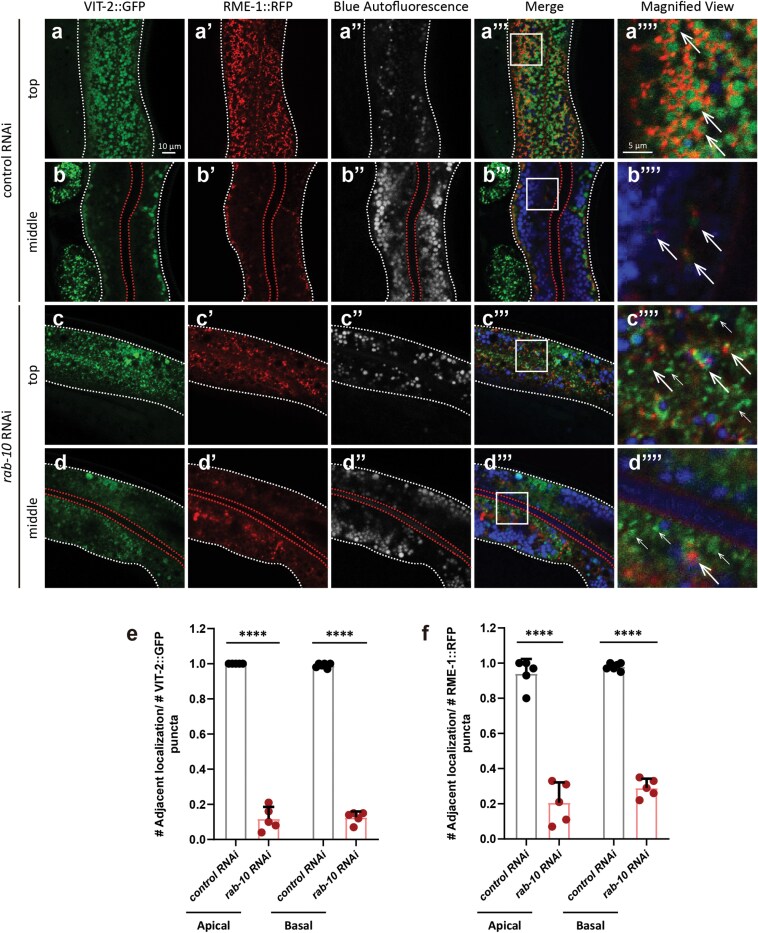
RAB-10 depletion disrupts the adjacency of RME-1::RFP puncta to VVs. (a–d) The basal and apical intestinal membranes are marked by white and red dashed lines, respectively. Thick white arrows indicate VIT-2::GFP puncta adjacent to RME-1::RFP puncta; thin arrows point to VIT-2::GFP puncta without adjacent RME-1::RFP. Rectangles are magnified and shown on the right. (e and f) Mean and SD. Each point represents a value from one region of one image (six images from six worms per group). ^****^P < 0.0001 by *t*-test analysis.

Given the above results, we next investigated the relationship between VVs, RME-1, and RAB-10. In *rab-10* knockdown worms, the VV periphery localization pattern of RME-1::RFP puncta was disrupted at both the basal side and apical side of the intestine (Fig. 9a−f). Moreover, RAB-10 depletion led to increased VIT-2::GFP signals at the apical side of the intestine and inversion of the asymmetric distribution of RME-1::RFP puncta in the intestine relative to that in non-targeted RNAi controls (Supplementary Fig. S4d and e). These results suggest that RAB-10 is required for RME-1::RFP localization at the VV periphery.

### Cell-autonomous regulation of VIT secretion in the *C. elegans* intestine

**Figure 10:**
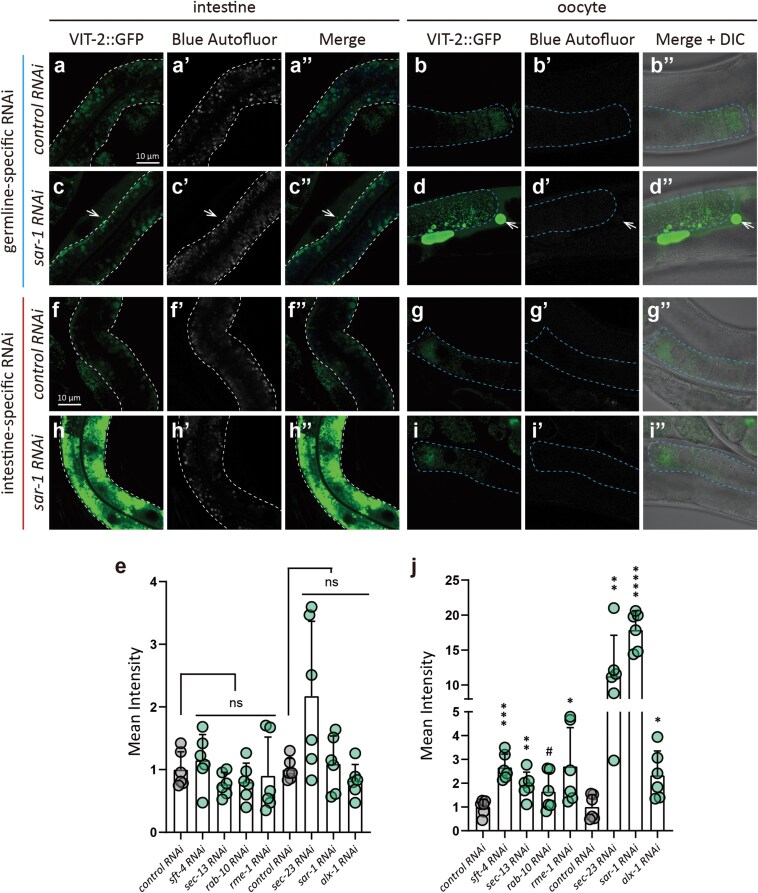
The tissue-specific RNAi verifies that the VIT-2::GFP phenotypes detected in the intestine are cell-autonomous. (a–d’’, f–i’’) The basal intestinal membrane and oocytes in the gonad are marked by white and blue dashed lines, respectively. White arrows indicate pseudocoelomic yolk. (e and j) Box plots showing the mean intensity of VIT-2::GFP in the intestine (mean and SD) after germline- and intestine-specific knockdown of genes. RNAi was performed since L4. Each point represents a value from one image (six images from six worms per group). ns, no significance; ^#^*P* < 0.1; ^*^P < 0.05; ^**^P < 0.01; ^***^P < 0.001; ^****^P < 0.0001 by *t*-test analysis.

We next sought to rule out the possibility that the intestinal VIT-2::GFP retention phenotype may result from the accumulation of yolk in the pseudocoelom that cannot be endocytosed by oocytes to compensate for RME-2 secretion [20, 61]. To this end, we used intestine- and germline-specific RNAi worm strains to determine whether candidate genes identified in our initial systemic RNAi screen could also affect VIT secretion in specific tissues. Confocal microscopy assays indicated that germline-specific RNAi of each conventional secretion pathway or endocytic recycling gene did not result in VIT-2::GFP retention in the intestine, although some germline-specific knockdown worms exhibited obvious accumulation of pseudocoelomic yolk (Fig. 10a–e; Supplementary Fig. S5). In contrast, intestine-specific RNAi of the same genes reproduced the intestinal VIT-2::GFP retention phenotype (Fig. 10f–j; Supplementary Fig. S6). These results thus led us to conclude that the knockdown-associated VIT-2::GFP retention phenotypes are in fact cell-autonomous.

## Discussion

### Interpretation of positive and negative RNAi results of VIT secretion

As individual proteins are often found to function in different biological processes, positive hits obtained by RNAi screening should be interpreted with caution. For example, CHC-1 regulates both clathrin-dependent endocytosis at the PM as well as secretory vesicle formation via budding from TGN [53, 62]. Therefore, intestinal VIT-2::GFP retention upon CHC-1 depletion might be due to defects in either early endosome-to-RE maturation or secretory vesicle budding at the TGN, or a combination of defects in these processes. Our findings that *chc-1* knockdown decreased the number of enlarged VIT-2::GFP-positive REs within the intestine of *rme-1* RNAi worms (Fig. 6c) could be interpreted to mean that *chc-1* is required for intestinal endocytosis of pseudocoelomic yolk proteins, or alternatively, that *chc-1* participates in delivering Golgi-derived VITs to REs.

We noted that the intestinal VIT retention phenotype is more prominent when knockdown is induced in L1 worms compared to knockdown at L4 (Supplementary Table S1), suggesting that some genes may indirectly affect VIT secretion. Moreover, intestinal VIT retention is more obvious in worms with knockdown of genes involved in upstream transport from ER to PM compared to knockdown of downstream genes (Supplementary Table S1). For example, depletion of ERES components caused the most severe intestinal VIT retention phenotype, while exocytosis or RE-associated gene knockdown conferred relatively weaker effects. These observations indicate that VITs may be synthesized in a specific organelle, that is, the ER, and then are likely to be delivered to a variety of compartments downstream of the secretion route.

Although 25 genes involved in the conventional secretion pathway showed positive phenotypes, 14 genes in this pathway had no apparent effect on VIT secretion, such as the AP-1 subunits, *aps-1*, *apb-1*, and *apm-1* (Supplementary Table S1), which are required for secretory vesicle budding from the TGN [51, 53, 63, 64]. The absence of VIT accumulation in these knockdown worms may be because these genes are simply not required for VIT secretion. Alternatively, the RNAi may have incompletely silenced these targets. However, it is also possible that more than one pathway can mediate VIT secretion, so blocking the conventional secretion pathway might result in shunting VIT secretion through another route.

### Endocytosis of pseudocoelomic substances occurs at the basal intestinal membrane

Although it may superficially appear paradoxical that intestines secrete VITs to the pseudocoelom while concurrently endocytosing cleaved VITs from the pseudocoelom, in *C. elegans,* the pseudocoelomic fluid functions similarly to circulating blood in vertebrates, mediating communication among different tissues through a diversity of signal molecules. To this end, the *C. elegans* intestine can endocytose pseudocoelomic substances secreted from other tissues [65–67], for instance, indiscriminately capturing GFP secreted from body wall muscles of *myo-3p::sel-1(signal sequence)::gfp* worms, or exogenously administered FM4-64 or double-stranded RNA (dsRNA) [27, 68]. After sexual maturity, the cleaved VITs, i.e. yolk proteins, are distributed widely throughout the pseudocoelom [22, 54, 69], and therefore, when the intestine takes up essential substances from the pseudocoelom, it inevitably takes up some of the mature yolk proteins. These inadvertently endocytosed yolk proteins can be delivered to lysosomes for degradation or exported again via REs [27].

### Communication between the TGN and endosomes

The results in this study suggest that the conventional secretion pathway is interconnected with endocytic recycling, in a manner dependent on communication between the TGN and endosomes [25, 26, 28, 70, 71]. Budding of clathrin-coated vesicles from the TGN is widely accepted to mediate cargo transport from TGN to endosomes [51], while the retromer complex promotes cargo delivery from endosomes to TGN in mammalian cells [71]. Silencing the only known GGA in *C. elegans*, the clathrin adaptor protein APT-9 [53], leads to VIT-2::GFP retention in the intestine (Fig. 4g) and can prevent formation of VIT-containing REs (Fig. 6c). These findings suggest that the mechanisms underpinning cargo delivery from TGN to endosomes may be conserved between *C. elegans* and mammals. However, additional experiments are necessary to determine whether the accumulated VIT-2::GFP is indeed trapped within the trans-Golgi cisterna of the intestine in *apt-9* RNAi worms.

### Transportation of newly synthesized VITs to REs

As we observed, blocking vesicle trafficking from the ER to the Golgi or from the TGN to endosomes can suppress the enhanced formation of VIT-containing REs caused by RME-1 depletion, suggesting that newly synthesized VITs can be transported into REs (Fig. 6a and c). However, the mechanism by which newly synthesized VITs are translocated into REs is not entirely understood. We hypothesized that TGN-derived VVs could fuse with REs, thereby depositing the VITs in REs. Exactly, a recently published study supports this hypothesis, and it revealed that RAB-10 and its effector EHBP-1 facilitate post-Golgi VV trafficking and tethering to REs in the *C. elegans* intestine [28]. Consistently, our immuno-EM data showed that membrane-less VIT-containing droplet structures are present inside enlarged REs in the intestines of *rme-1* RNAi worms. We also noted that such droplets shared similar immuno-EM characteristics with the contents of VVs, together supporting a scenario in which TGN-derived VVs may fuse with REs. However, these immuno-EM data cannot rule out the possibility that the contents of VVs come from the pseudocoelom, meaning that the contents of VVs might be secreted to the pseudocoelom, and then immediately endocytosed back into the intestine, where they subsequently display as droplet structures that are again packaged into REs.

### Are VVs *per se* intestinal REs or just adjacently localized with REs?

RME-1 is widely accepted as a marker of REs [27], and previous studies have shown that other regulators of endocytic recycling can physically interact or colocalize with RME-1 in *C. elegans* [49, 72]. For example, SDPN-1 (a Syndapin/PACSIN-family protein) was found to colocalize with RME-1 on REs and promote membrane tubulation by coordinating membrane-associated actin dynamics [45, 46]. Similarly, EHBP-1 can colocalize with RME-1 and together participate in bridging filamentous actin with tubular REs to facilitate endocytic recycling [35, 73]. RTKN-1 (Rhotekin) can also colocalize with RME-1 and SDPN-1 to facilitate basolateral endocytic recycling in the intestine by inhibiting endosome-associated F-actin disassembly [47]. Physical interaction between ALX-1 (an Alix/Bro1p family protein) and RME-1 has been demonstrated to be essential for basolateral endocytic recycling in the nematode intestine [39], while the interaction of AMPH-1 (the only *C. elegans* Amphiphysin/BIN1 family protein) with RME-1 leads to the cooperative promotion of cargo exit from REs to PM via endosomal tubulation [49].

As nearly all intestinal RME-1::RFP puncta are localized at the periphery of VVs in our study (Fig. 7a–c), and VVs are adjacently localized with the other two markers of REs, LET-423 (homologue of Scrib/Erbin) and hTAC (human interleukin-2 receptor alpha-chain), in the intestine of *C. elegans* [28], we sought to determine whether VVs *per se* formed intestinal REs or were localized in close proximity to REs. As no other membrane-bound structures were localized in the close vicinity of VVs in careful scrutiny of our immuno-EM and conventional EM data, we were inclined towards the former view.

### RAB-10 may indirectly recruit RME-1 to VVs

In the present study, RAB-10 depletion disrupted the localization of RME-1::RFP puncta at the VV periphery, indicating that RAB-10 is required for RME-1 recruitment to the VV membrane. This finding led us to ask how RAB-10 might affect the localization of RME-1 puncta near VVs. Previous reports suggest that RAB-10 functions upstream of RME-1 in basolateral endocytic recycling of the *C. elegans* intestine, and fluorescence microscopy assays have shown that only a small portion of RAB-10 puncta colocalize with RME-1 puncta in the intestine [37]. Based on this prior knowledge, we hypothesized that RAB-10 may indirectly recruit RME-1 to VVs.

Endocytosis-related studies have demonstrated that RAB-10 interacts with EHBP-1 through its C-terminal domain in *C. elegans* [28, 73]. In contrast, the mammalian homolog of RME-1, Ehd-1, can bind directly to the EHBP-1 homolog, Ehbp1, in mammals [35, 59]. In *C. elegans*, EHBP-1 colocalizes with, but does not directly bind to, RME-1 due to the absence of an asparagine-proline-phenylalanine (NPF) motif on EHBP-1 [73]. RNAi of *ehbp-1* phenocopies *rab-10* RNAi and *rme-1* RNAi, suggesting that RAB-10, EHBP-1, and RME-1 might coordinately function in basolateral endocytic recycling in the *C. elegans* intestine [27, 35, 39, 49, 73, 74]. These findings collectively indicate that RAB-10 recruits RME-1 to REs in an EHBP-1-dependent manner. Shi *et al*. described a relatively clear mechanism by which RAB-10 can indirectly recruit RME-1 to REs [43], wherein GTP-bound RAB-10 activates phosphatidylinositol-4-phosphate 5-kinase to promote the conversion of phosphatidylinositol 4-phosphate to phosphatidylinositol 4,5-bisphosphate. This lipid conversion subsequently facilitates the localization of RME-1 to the RE membrane [43]. However, more detailed studies in future research are needed to determine whether the same mechanisms also operate on the VV membrane.

### Potential mechanistic connection between VIT secretion in *C. elegans* and lipid transport in humans

VITs share a highly conserved lipid-binding domain, vitellogenin_ N,with two human proteins—apoB-100 and microsomal triglyceride transfer protein (MTP) [2, 75]. The apoB-100 and MTP proteins are both expressed in the liver. MTP facilitates the lipidation of apoB-100 within the ER, leading to the formation of VLDL particles [12, 13]. These VLDL particles are subsequently secreted by the liver into the bloodstream, transporting lipids synthesized in the liver to various tissues. As the circulating VLDL particles gradually lose their triglyceride load to hydrolysis catalyzed by lipoprotein lipase on the surface of vascular endothelium, they become low-density or intermediate-density lipoproteins, both of which can be recycled by the liver through endocytosis mediated by apoB-100 receptor or apoE receptor, respectively [12, 13, 76, 77].

Dysregulation of VLDL secretion from the liver is a direct cause of hepatic steatosis and atherosclerosis [12, 13, 78, 79]. Given the sequence conservation and the shared biochemical function among the vitellogenin_N-containing apolipoproteins, understanding the secretion mechanism of the lipoprotein assemblies of nematode VITs is likely to provide insights into the secretion process of VLDL particles in humans.

### Limitations of the study

This study found that REs facilitate the secretion of VITs/yolk proteins out of the *C. elegans* intestinal cells, but the detailed molecular mechanisms underlying this process are not investigated. Additionally, it is unknown whether this mechanism is conserved in humans, that is, whether REs in the liver facilitate secretion of VLDL assembled by apoB-100, the mammalian homologue of VITs/yolk proteins.

## Materials and methods

### Worm culture and worm strains

Worms were cultured on nematode growth medium (NGM) with *Escherichia coli* OP50 at 20°C. Worm strains used in this study are listed in Table 1.

**Table 1.** Worm strains used in this study.

No.	Strain name	Genotype
1	N2	*Caenorhabditis elegans* Genetics Center
2	BCN9071	*vit-2(crg9070[vit-2::gfp]) X*
3	MQD2947	*vit-6(hq486[vit-6::mCherry]) IV; hjIs14[vha-6p::gfp::C34B2.10 + unc-119(+)]*
4	MQD3015	*vit-6(hq486[vit-6::mCherry]) IV; rab-10(qxIs195[Pges-1::gfp::rab-10])*
5	MQD3016	*vit-2(crg9070[vit-2::gfp]) X; rab-5(pwIs846[Pvha-6::rfp::rab-5])*
6	MQD3018	*vit-2(crg9070[vit-2::gfp]) X; rme-1(qxIs216[Pvha-6::rme-1::rfp])*
7	MQD3072	*rde-1(mkcSi13[sun-1p::rde-1::sun-1 3’UTR + unc-119(+)]) II; rde-1(mkc36) V; vit-2(crg9070[vit-2::gfp]) X*
8	MQD3073	*rde-1(mkc36) V; vit-2(crg9070[vit-2::gfp]) X; rde-1(kbIs7[nhx-2p::rde-1 + rol-6(su1006)]*
9	MQD3280	*rme-1(hq635) V; vit-2(crg9070[vit-2::gfp]) X*
10	MQD3281	*rme-1(hq636) V; vit-2(crg9070[vit-2::gfp]) X*
11	MQD3282	*rme-1(hq637) V; vit-2(crg9070[vit-2::gfp]) X*
12	MQD3283	*rme-1(hq638) V; vit-2(crg9070[vit-2::gfp]) X*

### Worm strain construction

We generated *rme-1* knockout mutants in the BCN9070 *vit-2(crg9070[vit-2::gfp])* background using CRISPR-Cas9 methods [80]. Four resulting strains (MQD3280, MQD3281, MQD3282, and MQD3283), which were used in Supplementary Fig. S3a–a’, contained frameshift mutations beginning in the 5^th^ exon of *rme-1* (W06H8.1a) (the mutated DNA sequences are listed in Supporting Files S1). The sgRNA target sequences for *rme-1* knockout were: 5’-TTTCGGAGAGCAGCGTTACG-3’ and 5’-ACAGGTCGTGCTGCTCGTCT-3’. Other strains were generated through genetic crosses.

### RNAi

RNAi-mediated knockdown was conducted by following the conditions described previously, except that 1 mmol/L isopropyl β-D-1-thiogalactopyranoside (IPTG) was used for induction [81]. Some target sequences were amplified via PCR from the *C. elegans* genome, and then inserted into to L4440 vector between T7 promoters, or directly extracted L4440 plasmid-carrying targets from HT1115 bacteria of Ahringer or Vidal bacterial libraries [82, 83], with more details shown in Supplementary Table S2. All constructed and extracted plasmids were transfected into iOP50 *[rnc14::ΔTn10 laczγA::T7pol camFRT]* for RNAi [84]. For RNAi of double genes, two bacteria with the same OD were mixed in equal volume. RNAi started with synchronized L1 or early L4 larvae. Adult day 1 (AD 1) or AD 2 adults were harvested for light or electron microscopy.

### Fluorescent microscopy imaging

A total of 25 worms at AD 1 were picked on a glass slide with a 3% agarose pad, and paralyzed with 10 mmol/L levamisole (Merck). Then, the samples were examined using NIKON A1R confocal microscope equipped with a 63× oil-immersion objective (magnification 630×). Pictures were visualized via NIS-Elements Viewer or ImageJ software. The experiments of quantifying the number of enlarged REs (Fig. 6b-d; Supplementary Fig. S3a–a’, f, g) were performed using the ZEISS LSM 880 microscope equipped with a 63× oil-immersion objective (magnification 630×). Pictures were visualized with the software Axio Vision Rel. 4.7.

### Quantification of intestinal VIT-2::GFP fluorescent intensity

The detailed quantification method is described in Supplementary Figure S1. Briefly, with ImageJ software, the intestinal area was circumscribed and divided longitudinally into apical and basal compartments. Images were converted to 8-bit format for analysis. Autofluorescent granules were segmented in the blue channel (405 nm) using a brightness threshold of 0–20. Subsequently, the green fluorescent signal (488 nm) was segmented using a brightness threshold of 25–255, with autofluorescent regions (identified in the previous step) masked by filling with black, as the gray values of the background were almost equal to 0 (Supplementary Fig. S1c). This procedure yielded specific VIT-2::GFP signal regions. Finally, both fluorescence intensity and intestinal area were measured. According to the functions listed in Supplementary Figure S1b, both the mean intensity of intestinal VIT-2::GFP and its intestinal distribution can be quantified.

### Immuno-EM

The process of sample preparation for immuno-EM has been well described before [22]. Here are the main experimental steps of the sample preparation. First, worms were fixed via high-pressure fixation (Wohlwend HPF Compact-01 apparatus), and then dehydrated via freeze substitution (Leica AFS2) with 1% paraformaldehyde in acetone. Second, the samples were filtered with LR White resin (London Resin, AGR1281A) at 4°C for 2 days. Finally, the samples were embedded in LR White resin containing 0.5% accelerator (London Resin, AGR1283) and polymerized at 4°C with UV irradiation.

EM samples were sectioned via a diamond knife (Diatome, DU4530) mounted on the Leica ultramicrotome (UC6, Leica, Germany). Immuno-EM sections were collected on Nickel grids and then incubated with anti-VIT-1/2 antibody (1:150, diluted in 1% BSA antibody buffer), which was kindly provided by Dr. Xiao-Chen Wang (Institute of Biophysics, CAS), for 1 h at room temperature [85]. The secondary antibody is a 10-nm gold conjugated anti-rat antibody (Sigma, SLBZ8963). The samples were incubated with the secondary antibody for 45 min at room temperature. Immuno-EM sections were imaged via Spirit T120 transmission electron microscope (FEI, USA) operating at 120 kV. Images were captured with a MoradeG3 CCD (EMSIS) camera using the RADIUS (EMSIS GmbH) software.

### Conventional EM

The workflow of sample preparation for conventional EM was clearly described before [86]. In this study, we made some modifications. Briefly, worms were fixed via high-pressure fixation (Wohlwend HPF Compact-01 apparatus), and then dehydrated via freeze substitution (Leica AFS2) with 1% OsO_4_ in acetone. SPI-PON 812 resin kit (SPI-CHEM) was used for filtration and embedding. Samples were polymerized at 60°C for 48 h. Sample blocks were sectioned via Leica ultramicrotome (UC6, Leica, Germany). The sections were collected on tapes and then mounted on SEM Cylinder Specimen Mounts (Electron Microscopy China) with carbon conductive double-faced adhesive tape (NISSHIN EM Co. Ltd., Japan). Sections were imaged via SEM (FEI Helios NanoLab 600i) equipped with a CBS detector. Images were acquired by the software xT microscope control (FEI, version 5.2.2.2898) with parameter settings of 2 kV accelerating voltage.

### Data analysis

In addition to the quantification of VIT-2::GFP intensity, other quantitative data were collected manually using ImageJ software. For the quantification of adjacency between RME-1::RFP puncta and VVs, the regions of interest (ROIs, each about 400 μm^2^) in each image were selected, and the number of adjacent colocalization events, the number of RME-1::RFP puncta, and the number of VIT-2::GFP-labeled VVs in the intestine were manually counted. For the quantification of colocalization between VIT-2::GFP and RFP::RAB-5, the ROIs (each about 300 μm^2^) in each image were selected, and the Pearson’s correlation coefficient was measured via the Coloc 2 plugin in the ImageJ software. The pixel size was determined via the scale bar labeled on the original images. The quantitative data were analyzed by GraphPad Prism 8.4.3. Values in each group were tested for normality via the Shapiro–Wilk test or the Kolmogorov–Smirnov test. If the data points were not normally distributed, the Mann–Whitney test was used for difference analysis between the two groups. If the data points were normally distributed, the unpaired *t*-test was used for difference analysis if variances were homogenous (*F*-test, *P* ≥ 0.05), or the Welch’s *t*-test was used if variances were not homogenous (*F*-test, *P* < 0.05). ^#^*P* < 0.1; ^*^P < 0.05; ^**^P < 0.01; ^***^P < 0.001; ^****^P < 0.0001. ns means no significance.

## Illustration

Figure 1a, Figure 1b, and Figure 6a were created using BioRender.

## Supplementary Material

loaf026_suppl_Supplementary_Figures_S1-S6_Tables_S1-S2

loaf026_suppl_Supplementary_Videos

## Data Availability

All data supporting the findings of this study are included in the supplementary materials and are available from the corresponding authors upon reasonable request. This work represents original research that has not been published previously and is not under consideration for publication elsewhere, in whole or in part.
