# Supplementary material for: RME-1-associated recycling endosomes participate in vitellogenin secretion in Caenorhabditis elegans
Source: Life Metab. 2025 Jun 28;4(6):loaf026. doi: 10.1093/lifemeta/loaf026 (PMC12507027; doi:10.1093/lifemeta/loaf026)
Supplement: loaf026_suppl_Supplementary_Figures_S1-S6_Tables_S1-S2 [file loaf026_suppl_supplementary_figures_s1-s6_tables_s1-s2.pdf]

a

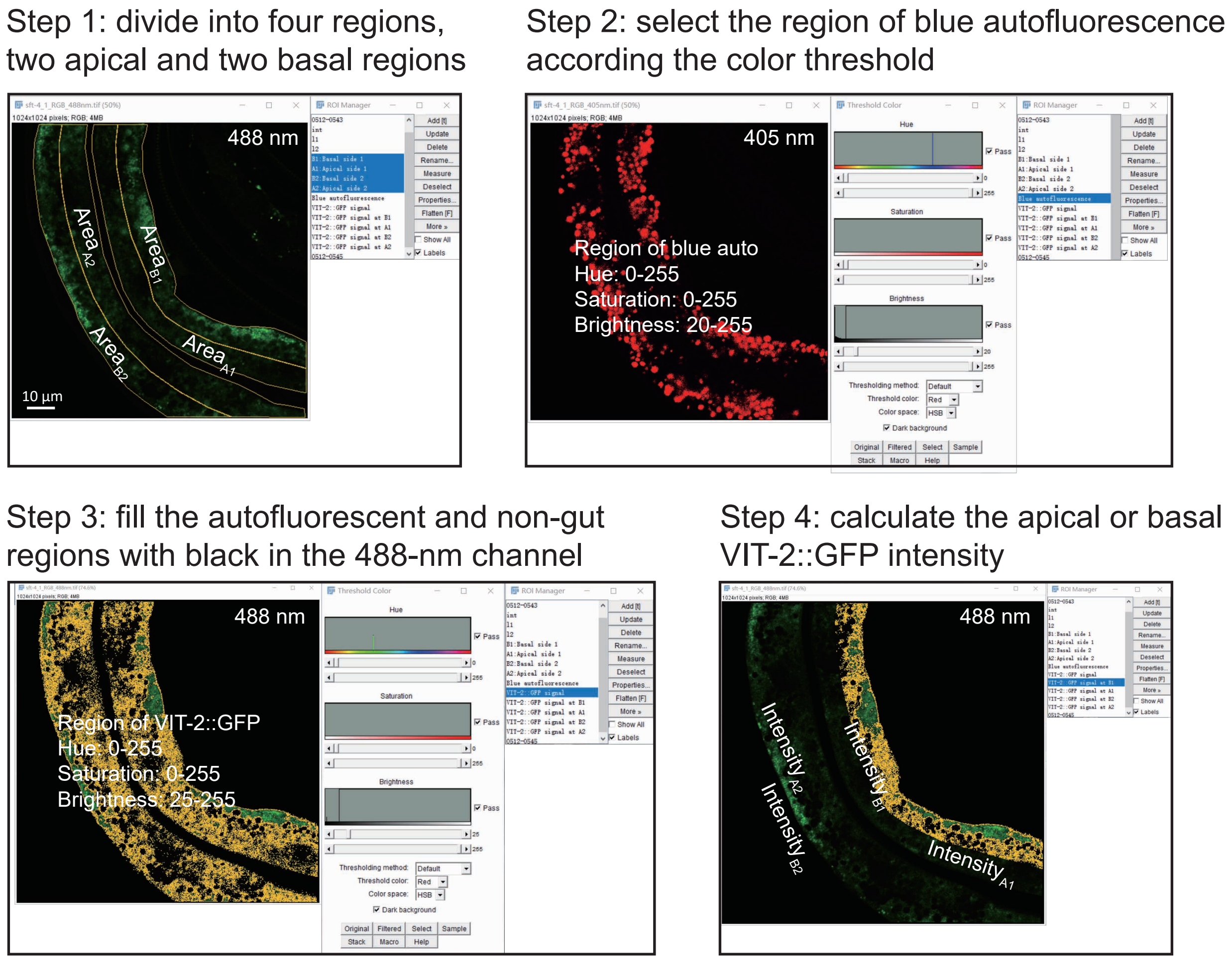

b

Mean intensity = $(\text{Intensity}_{B1} + \text{Intensity}_{A1} + \text{Intensity}_{B2} + \text{Intensity}_{A2}) / (\text{Area}_{B1} + \text{Area}_{A1} + \text{Area}_{B2} + \text{Area}_{A2})$

Apical intensity = $(\text{Intensity}_{A1} + \text{Intensity}_{A2}) / (\text{Area}_{A1} + \text{Area}_{A2})$

Basal intensity = $(\text{Intensity}_{B1} + \text{Intensity}_{B2}) / (\text{Area}_{B1} + \text{Area}_{B2})$

Percentage of Apical intensity = Apical intensity/ (Apical intensity + Basal intensity)

Percentage of Basal intensity = Basal intensity/ (Apical intensity + Basal intensity)

C

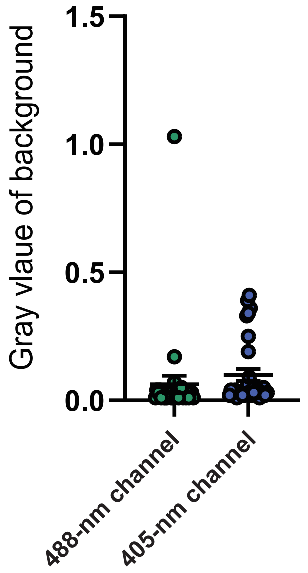

Supplementary Figure S1 Workflow for quantifying intestinal VIT-2::GFP fluorescence intensity. (a) Image processing steps using ImageJ: Import the image of the 488-nm channel, showing VIT-2::GFP and gut granule autofluorescence (gut granules also emit blue autofluorescence in the 405-nm channel). Divide the intestine into four regions (two apical, two basal) along the midline and calculate their areas (AreaB1, AreaA1, AreaB2, and AreaA2). Import the 405-nm channel image, and select blue autofluorescence regions using color threshold (Hue: 0–255, Saturation: 0–255, Brightness: 20–255). Re-import the 488-nm channel image, mask autofluorescence, and non-intestinal regions with black (background gray value ≈ 0). Select VIT-2::GFP signal using color threshold (Hue: 0–255, Saturation: 0–255, Brightness: 25–255). Divide intensity into four parts (intensityB1, intensityA1, intensityB2, and intensityA2) based on intestinal regions. (b) Functions to calculate the mean intensity of VIT-2::GFP and the percentage of apical or basal intensity of VIT-2::GFP, reflecting accumulation and distribution, respectively. Six images (six worms) per sample. (c) The dot plot shows background gray values of confocal images. Each point represents one value from a randomly selected background region (five regions per image, six images from six worms for each channel).

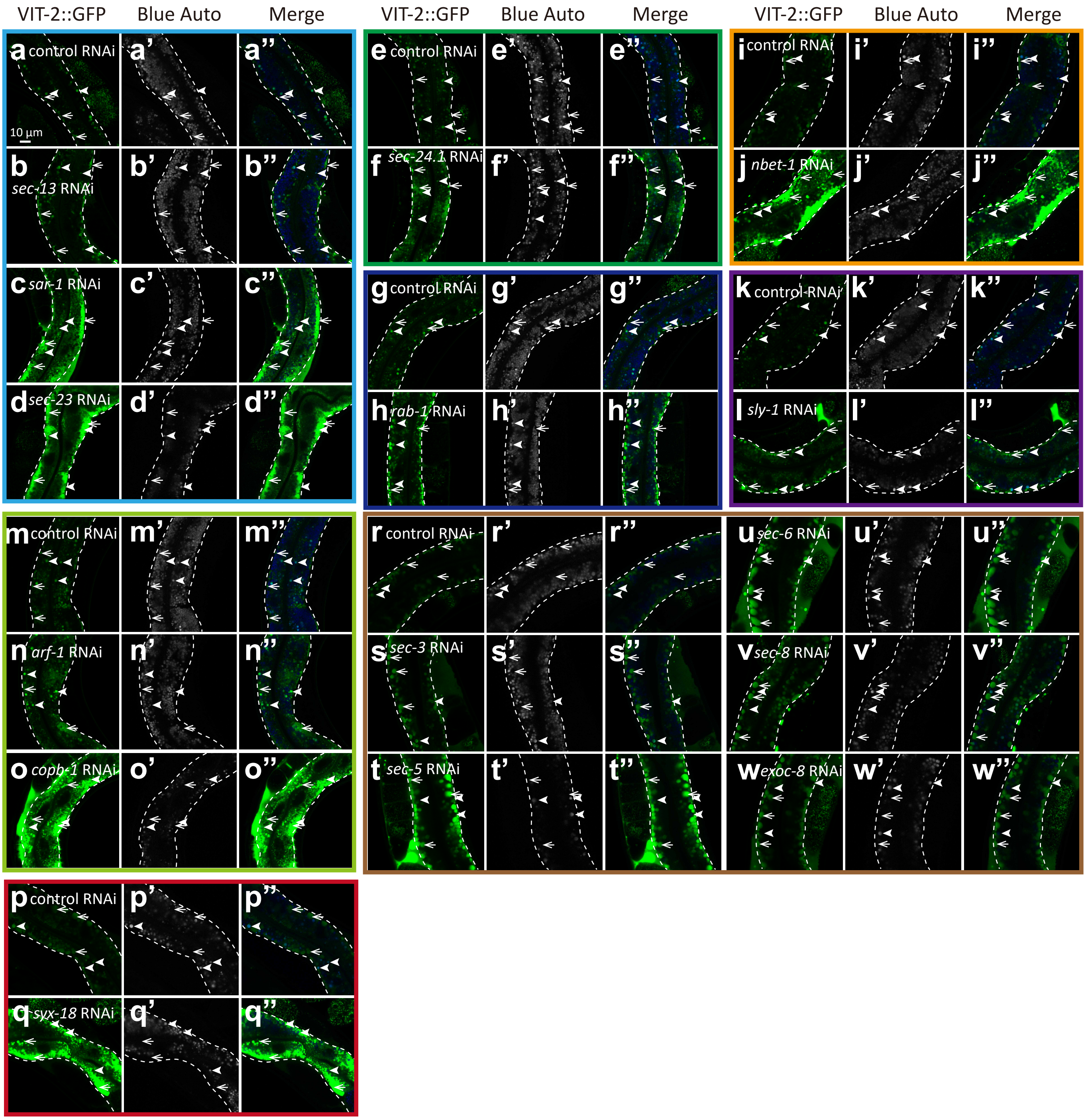

Supplementary Figure S2 Silencing genes involved in the conventional secretion pathway causes VIT-2::GFP accumulation in the intestine. Fluorescent images of *vit-2::gfp* knock-in worms at AD 1. Arrows point to VIT-2:::GFP, and arrowheads point to gut granules emitting green and blue autofluorescence (Auto). Pictures in the same rectangle are captured in one trial. White dashed lines profile the intestine.

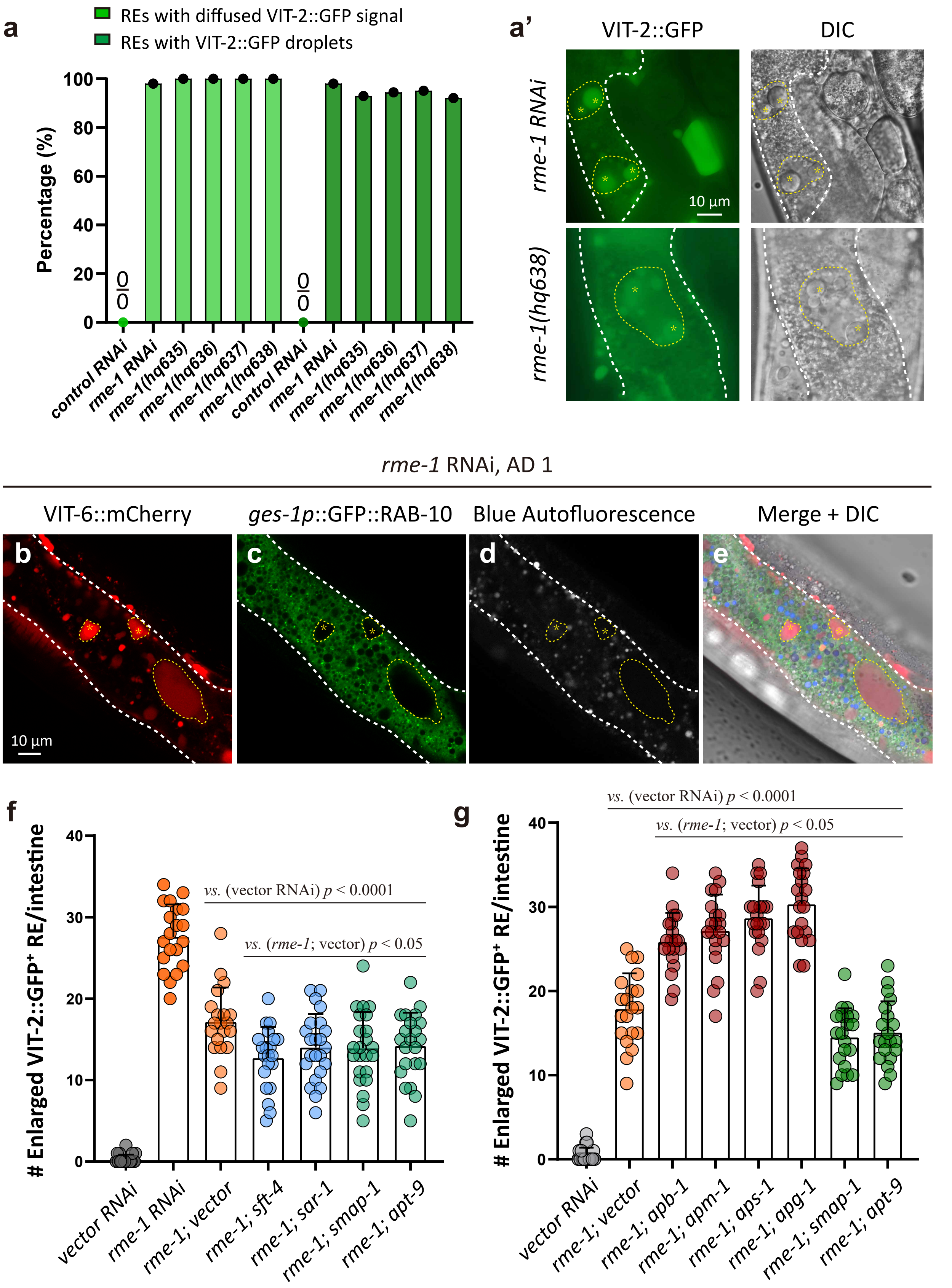

Supplementary Figure S3 Enlarged REs contain diffused VIT signal and VIT droplets upon RME-1 depletion, and (f and g) are experimental repeats of Figure 6c and d. (a and a') Analysis of > 41 REs from 15–30 worms per group, examined at AD 1. A total of 25 worms of control RNAi were examined, and nearly no enlarged RE was detected. (b–e) VIT-2::GFP or VIT-6::mCherry droplets within enlarged REs (yellow dashed lines) marked by *Asterisks. White dashed lines outline the intestine. (f and g) Box plots showing the mean and SD. Each point represents RE counts from one intestine, and 20 intestines were analyzed for each bar. (a–g) *rme-1* RNAi since L1; RNAi of other genes since early L4. *t*-test analysis.

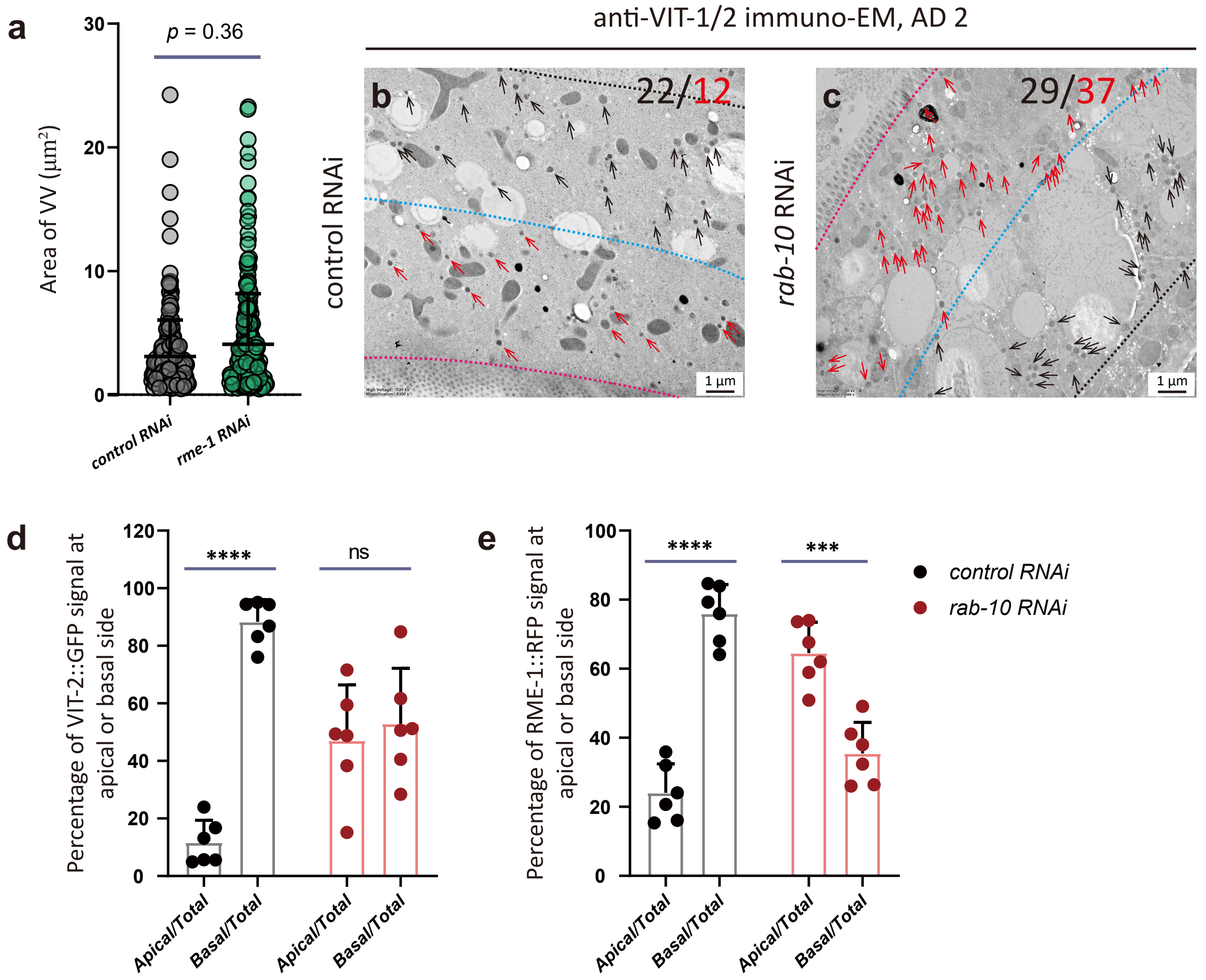

Supplementary Figure S4 RAB-10 depletion alters the distribution of VVs and RME-1::RFP in the intestine. (a) VV size measured based on fluorescent images of *vit-2::gfp* KI worms (at AD 1) treated with RNAi since L1. six images of six worms were analyzed to measure the size of VVs. The dot plot shows the Mean and SD. Each point means one value of a VV. Mann-Whitney test. (b and c) The basal and apical intestinal membranes marked by black and red dashed lines, respectively. Blue dashed lines divide the intestine into apical and basal regions for quantifying VV distribution. Black arrows indicate basal VVs; red arrows indicate apical VVs. Counts are labeled in corresponding colors on the top right. (d and e) Mean and SD. Each point represents a value from one image of a *vit-2::gfp;vha-6p::rme-1::rfp* worm (six images from six worms per group). ns, no significance; $^{***}P < 0.001$; $^{****}P < 0.0001$ by *t*-test analysis.

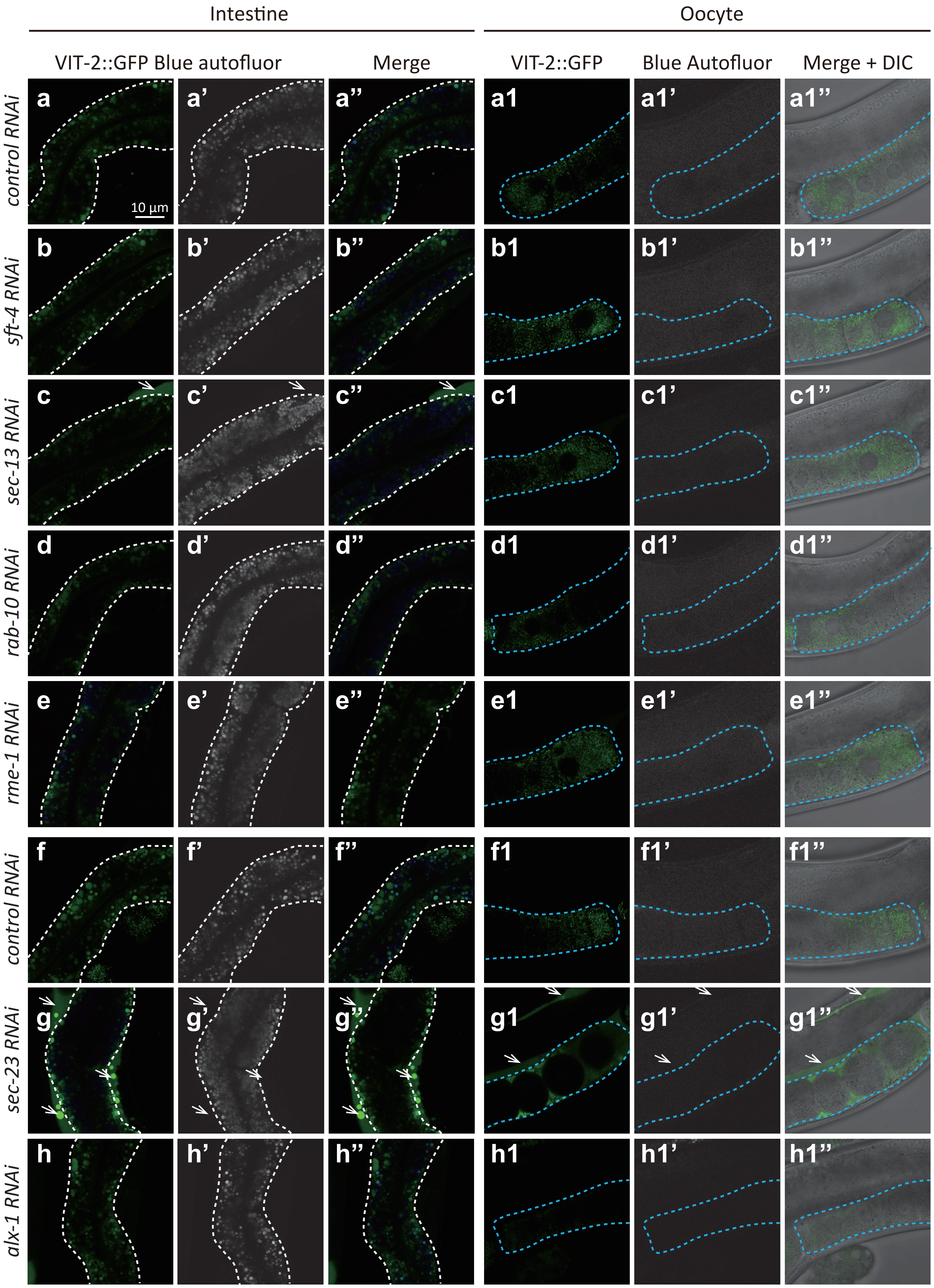

Supplementary Figure S5 Germline-specific RNAi of genes involved in the conventional secretion pathway and endocytic recycling. (a–h1’’) The basal membrane of the intestine and oocytes in the gonad indicated by white dashed lines and blue dashed lines, respectively. (c–c’’ and g–g1’’) Pseudocoelomic yolk indicated by white arrows. RNAi was conducted since early L4 and worms were examined at AD 1.

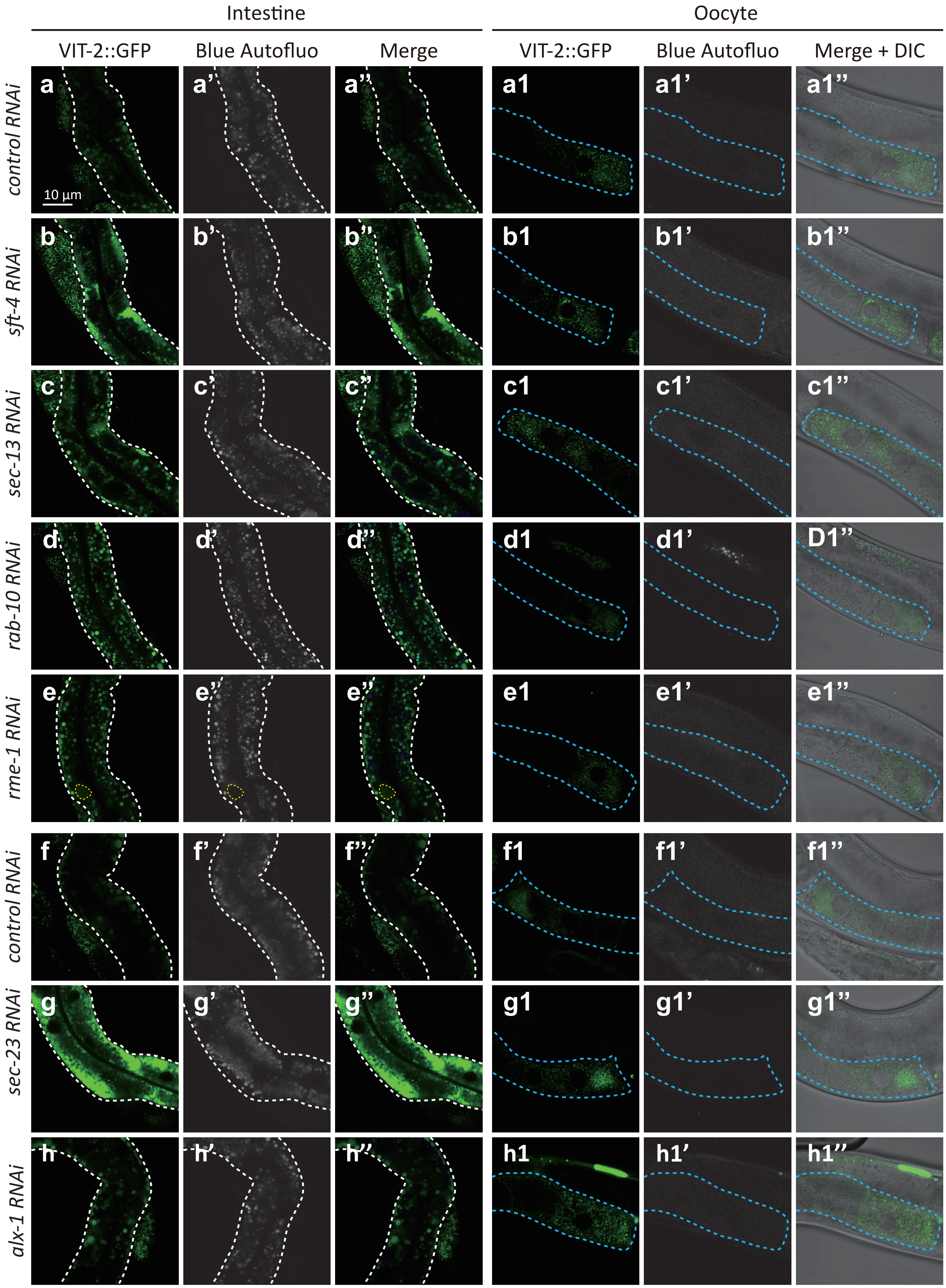

Supplementary Figure S6 Intestine-specific RNAi of genes involved in the conventional secretion pathway and endocytic recycling. (a–h1'') The basal membrane of the intestine and oocytes in the gonad indicated by white dashed lines and blue dashed lines, respectively. (e–e'') The yellow dashed line profiles an enlarged RE in the intestine. RNAi was conducted since early L4 and worms were examined at AD 1.

Supplementary Table S1 Results of RNAi screen.

Fed *vit-2::gfp* KI worms with iOP50 RNAi bacteria since L1 or L4, and examine the worms on adult day 1.
"-" means no change, "+" means the intensity or localization of intestinal VIT-2::GFP changed slightly (+) or greatly (++), and "/" means different biological repeats. “N" means did not test.

Number	Gene	Seqence	Function	RNAi since L1	RNAi since L4	Intestinal phenotype
1	*sft-4*	C54H2.5	Assembly of ER exit sites	++/++	++	VIT-2::GFP spots gathered at the basal side of the intestine.
2	*sec-13*	Y77E11A.13a	Assembly of ER exit sites	++	+	VIT-2::GFP signal accumulated in the intestine.
3	*sar-1*	ZK180.4	Assembly of ER exit sites	arrested and die at L1	++	VIT-2::GFP spots gather like patches.
4	*sec-24.1*	F12F6.6	Assembly of ER exit sites	arrested and die at L2	++	-
5	*sec-23*	Y113G7A.3	Assembly of ER exit sites	arrested and die at L3	++	Numerous GFP spots localized at the basal side of the intestine.
6	*sly-1*	F43D9.3	Cargo entrance to *cis* -Golgi	arrested at L2 or L3	++	Many weak GFP spots accumulated in the intestinal cells, nearly no bigger VVs.
7	*syx-5*	F55A11.2	Cargo entrance to *cis* -Golgi	++	++	Less GFP signal in the thin intestine
8	*uso-1*	K09B11.9	Cargo entrance to *cis* -Golgi	++	+	Numerous weak GFP spots accumulated in the intestine. No normal size VVs.
9	*rab-1*	C39F7.4	Cargo entrance to *cis* -Golgi	arrested at L1	++	Nomal size VVs and smaller VVs are accumulated in the intestine.
10	*trpp-1*	DC2.8	Cargo entrance to *cis* -Golgi		-	-
11	*trpp-3*	ZK1098.5	Cargo entrance to *cis* -Golgi	-	-	-
12	*trpp-4*	F36D4.2	Cargo entrance to *cis* -Golgi	-	-	-
13	*trpp-5*	Y57A10A.16	Cargo entrance to *cis* -Golgi	+	-	-
14	*trpp-6*	K08H10.9	Cargo entrance to *cis* -Golgi	-	-	-
15		Y42H9AR.1	Cargo entrance to *cis* -Golgi	-	-	-
16	*golg-2*	F33G12.5	Cargo entrance to *cis* -Golgi		-	-
17	*sql-1*	Y111B2A.4	Cargo entrance to *cis* -Golgi	-	-	-
18	*nbet-1*	Y59E9AL.7	Cargo entrance to *cis* -Golgi	++	N	More VIT-2::GFP spots localized in the intestine, and the VIT-2::GFP intensity increased greatly.
19	arf-1	B0336.2	Retrograde transport from Golgi to ER		++	More VIT-2::GFP spots localized in the intestine
20	*copb-1*	Y25C1A.5	Retrograde transport from Golgi to ER	arrested and die at L1	++	Bigger VVs gathered like patches at intestinal cell-cell interacted sites.
21	*syx-18*	T10H9.3	Retrograde transport from Golgi to ER	++	N	More VIT-2::GFP spots localized in the intestine, and the VIT-2::GFP intensity increased greatly.
22	*rab-18*	Y92C3B.3	Predicted to localize in the Golgi	-	-	-
23	*rab-6.1*	F59B2.7	Predicted to localize in the Golgi	-	-	-
24	*trpp-9*	C35C5.6	*trans* -Golgi cargo exit	N	-	-
25	*trpp-10*	Y71G12A.2	*trans* -Golgi cargo exit	N	-	-
26		*C56C10.7*	*trans* -Golgi cargo exit	N	+	More VIT-2::GFP spots localized in the intestine
27	*aps-1*	F29G9.3	*trans* -Golgi cargo exit	slim adults	-	-
28	*apb-1*	Y71H2B.10	*trans* -Golgi cargo exit	arrested at L3-L4 stages	-	-
29	*apm-1*	F55A12.7	*trans* -Golgi cargo exit	arrested at L3-L4 stages	-	-
30	*apg-1*	Y105E8A.9	*trans* -Golgi cargo exit	arrested at L3-L4 stages	-	-
31	*smap-1*	W09D10.1	*trans* -Golgi cargo exit	+	+	More VIT-2::GFP spots localized in the intestine
32	*apt-9*	W04G3.4	*trans* -Golgi cargo exit	+	+	More VIT-2::GFP spots localized in the intestine.
33	*chc-1*	T20G5.1	*trans* -Golgi cargo exit	arrested at L1	++	-
34	*sec-8*	Y106G6H.7	Exocyst subunits	+/ -	+	Small GFP-positive spots are distributed in the entire cytoplasm of the intesinal cells.
35	*exoc-7*	C43E11.8	Exocyst subunits	++/++	+	Small GFP-positive spots are distributed in the entire cytoplasm of the intesinal cells.
36	*sec-5*	T23G7.4	Exocyst subunits	++/++	-	Nearly all VVs localized at the basal membrane of the intestine.
37	*sec-3*	F52E4.7	Exocyst subunits	+/-	-	-
38	*exoc-8*	Y105E8B.e	Exocyst subunits	+	+	-
39	*sec-10*	C33H5.9	Exocyst subunits	-/+	+	No obvious VVs, but GFP localized on a net-like structures in the intesitnal cells.
40	*sec-15*	C28G1.3	Exocyst subunits	-	-	-
41	*sec-6*	F09E5.5	Exocyst subunits	+/+	-	-
42	*rab-11.1*	F53G12.1	Endocytotic recycling		-	-
43	*rab-11.2*	W04G5.2	Endocytotic recycling	++	++	Smaller VVs are accumulated in the intestine.
44	*rab-8*	D1037.4	Endocytotic recycling	-	-	-
45		F54C9.11	Endocytotic recycling	-	-	-
46	*rab-35*	Y47D3A.25	Endocytotic recycling	N	+	More VIT-2::GFP spots localized in the intestine
47	*rab-10*	T23H2.5	Endocytotic recycling	++/++	+	The GFP signal localizes at the apical side of the intestine. Big vacuole-like structures in the intestine.
48	*amph-1*	F58G6.1	Endocytotic recycling	-/-	-	-
49	*sdpn-1*	F45E1.7	Endocytotic recycling	-	-	-
50	*alx-1*	R10E12.1	Endocytotic recycling		+	More VIT-2::GFP spots localized in the intestine
51	*rme-1*	W06H8.1	Endocytotic recycling	+/+	+	Enlarged recycling endosomes can be detected and they have the VIT-2::GFP signal inside
52	*snap-29*	K02D10.5	Endocytotic recycling	arrested at L3-L4 stages	++	Dispersed VIT-2::GFP signal is distributed the whole cytoplasm of the intestine
53	*ehbp-1*	F25B3.1	Endocytotic recycling	-	N	-
54	*arf-6*	Y116A8C.12	Endocytotic recycling	-	N	-
55	*sid-3/kin-25*	B0302.1	Endocytotic recycling	-	N	-
56	*sac-1/sacm-1L*	F30a10.6	Endocytotic recycling	-	N	-
57	*ptrn-1/pqn-34*	F35B3.5	Endocytotic recycling	-	N	-
58	*rtkn-1*	C07B5.4	Endocytotic recycling	-	N	-
59	*syx-17*	VF39H2L.1	Regulated secretion	-	-	N
60	*snt-4*	T23H2.2	Regulated secretion	-/-	-	N
61	*snt-2*	F42G9.7	Regulated secretion	-	-	N
62	*rab-27*	Y87G2A.4	Regulated secretion	-	-	N
63	*rab-3*	C18A3.6	Regulated secretion	-	-	N
64	*unc-64/syx-1*	F56A8.7	SNARE protein, on basolateral plasma membrane	-	N	-
65	*syn-1/syx-3*	F35C8.4	SNARE protein, on plasma membrane	-	N	-
66	*syx-6*	C15C7.1	SNARE protein, on Golgi trans cisterna	-	N	-

67	*syn-13/syx-7*	F36F2.4	SNARE protein, on membrane and synaptic vesicle	-	N	-
68	*ric-4/snap-25*	Y22F5A.3	SNARE protein, on plasma membrane and synapse	-	N	-
69	*aex-4*	T14G12.2	SNARE protein, on plasma membrane	+/-	N	The VIT-2::GFP intensity increased.
70	*snb-7*	ZK795.4	SNARE protein, on plasma membrane	-	N	-
71	*snb-5*	C30A5.5	SNARE protein, on plasma membrane	-	N	-
72	*snb-6*	T14D7.3	SNARE protein, on plasma membrane	-	N	-
73	*vamp-8*	B0513.9	SNARE protein, on membrane	-	N	-
74	*vamp-7*	Y69A2AR.6	SNARE protein, on plasma membrane	-	N	-
75	*gos-28/gosr-1*	F08F8.8	SNARE protein, on Golgi medial cisterna	-	N	-
76	*ykt-6*	B0361.10	Cargo entrance to *cis*-Golgi	++	+	In addition to normal VVs, there are many smaller GFP spots gathering at the basal side of the intesine.
77	*snt-6*	C08G5.4	Synaptotagmin-VII	+	N	The VIT-2::GFP intensity increased slightly.
78	*cup-5/muc-1*	R13A5.1	TRPML1	+	N	The VIT-2::GFP intensity increased slightly.
79	*unc-116*	R05D3.7	KIF5B	+	N	The VIT-2::GFP intensity increased slightly.

Supplementary Table S2 Worm strains used in this study.

NO.	Strain name	Genotype
1	N2	*Caenorhabditis elegans* Genetics Center
2	BCN9071	*vit-2(crg9070[vit-2::gfp]) X*
3	MQD2947	*vit-6(hq486[vit-6::mCherry]) IV; hjIs14[vha-6p::gfp::C34B2.10 + unc-119(+)]*
4	MQD3015	*vit-6(hq486[vit-6::mCherry]) IV; rab-10(qxIs195[Pges-1::gfp::rab-10])*
5	MQD3016	*vit-2(crg9070[vit-2::gfp]) X; rab-5(pwIs846[Pvha-6::rfp::rab-5])*
6	MQD3018	*vit-2(crg9070[vit-2::gfp]) X; rme-1(qxIs216[Pvha-6::rme-1::rfp])*
7	MQD3072	*rde-1(mkcSi13[sun-1p::rde-1::sun-1 3'UTR + unc-119(+)]) II; rde-1(mkc36) V; vit-2(crg9070[vit-2::gfp]) X*
8	MQD3073	*rde-1(mkc36) V; vit-2(crg9070[vit-2::gfp]) X; rde-1(kbIs7[nhx-2p::rde-1 + rol-6(su1006)]*
9	MQD3280	*rme-1(hq635) V; vit-2(crg9070[vit-2::gfp]) X*
10	MQD3281	*rme-1(hq636) V; vit-2(crg9070[vit-2::gfp]) X*
11	MQD3282	*rme-1(hq637) V; vit-2(crg9070[vit-2::gfp]) X*
12	MQD3283	*rme-1(hq638) V; vit-2(crg9070[vit-2::gfp]) X*

Supplementary Table S3 Bacterial colonies for RNAi.

NO	Gene	Seqence	target sequence (5'-flank)	target sequence (3'-flank)	Source
1	*sft-4*	C54H2.5	TCAGAACGAAATGCTGGCGAAAGC	TCTTGTAGTCATCGACTGACACTCCTC	Conctructed
2	*sec-13*	Y77E11A.13a	GCAATTGTAAGTCGGAAAATCTG	TGAGAAAATTGCGGTTTTTGTAT	Ahringer library
3	*sar-1*	ZK180.4	ATTCAATATGTCGTTTTTATGGGAC	ATGGGGATGTTTGAATGATAGTGATG	Ahringer library
4	*sec-24.1*	F12F6.6	ATTCGACTGCATGGCAAGAGTAAGAACGTC	TATTATTTTGATCACGCCAAAGCAAATCTT	Ahringer library
5	*sec-23*	Y113G7A.3	CTACAGCCAATCAGTGAATGTGATGAATCG	TACGTACGGAGAGCAGAAGCTGACTACAAA	Ahringer library
6	*sly-1*	F43D9.3	AAGCAACAGTTAACACTATACATGC	TTCAGATACTCTAGATTCCAATCAATTAC	Conctructed
7	*syx-5*	F55A11.2	TTCAGCAGTTGCTCCGACTA	TGACAGAGCACCCATATCCA	Ahringer library
8	*uso-1*	K09B11.9	TTTTCGATTTTTCGATTTTCTGA	GCAACTTCTTCGAGTAAACCTCA	Ahringer library
9	*rab-1*	C39F7.4	ATGGGCTCTCGTGACGATGAATACGACTAC	GCGGTGGCAGTGGTACAATCATTCCTTCGC	Conctructed
10	*trpp-1*	DC2.8	ATGACGATTTACAACGTGTACATATTCGAC	CTGACAGTCTGATTAGAATCATTGGTTGC	Conctructed
11	*trpp-3*	ZK1098.5	CCTTAAAATCGGTCTCTTTCGTT	AATGAGAAAATTTGAACAAGCCA	Ahringer library
12	*trpp-4*	F36D4.2	cDNA		RCE library
13	*trpp-5*	Y57A10A.16	cDNA		RCE library
14	*trpp-6*	K08H10.9	ATACTTCGTCCCCGTGTTTG	GCTCCACCAAATTTCCAAA	Ahringer library
15		Y42H9AR.1	cDNA		RCE library
16	*golg-2*	F33G12.5	TCAGTGATATGGTCGATGCCGCTG	GTTTGACGAAAGTCCAGATGATGTTGA	Conctructed
17	*sql-1*	Y111B2A.4	cDNA		RCE library
18	*nbet-1*	Y59E9AL.7	ATGGCATTTCGAGGTGGTCAAATGCGAGGT	AAGATTACACTCAATCGAAAACTGCTCGTT	Ahringer library
19	arf-1	B0336.2	TTGAGAGAGGAAGAGACACTCCACTGGTCG	CACCTTCGACGAATAAACGACTACGAATCA	Ahringer library
20	*copb-1*	Y25C1A.5	TTCACAAAAACACATTTTATGGCAAAAAAT	GGAGAATGGTGTGATCTTTTCAACGATTTC	Ahringer library
21	*syx-18*	T10H9.3	ATCGTCCATCTCCAGCATTCACCAACATTT	GAGATTCAAGCAAAGCACCCATGTATGCAG	Ahringer library
22	*rab-18*	Y92C3B.3	cDNA		RCE library
23	*rab-6.1*	F59B2.7	AGGATTCATGATAACGATGTTGG	AAATTTTACGCAGAAATGCAGAA	Ahringer library
24	*trpp-9*	C35C5.6	AAGGAGGTCCGCCACTTATT	TCTTCTTCGCACCGGTAACT	Ahringer library
25	*trpp-10*	Y71G12A.2	TTTCCCAATTTGAATACCCG	TCACTTTTGCGAACCAATC	Ahringer library
26		*C56C10.7*	AGTCTGCAAGACACTTCAAAAGG	CTCTGCGGGGTAATTTTAGTTCT	Ahringer library
27	*aps-1*	F29G9.3	ACGAAGCCACATGTCTGTTTTAT	TTGGTATTTACGGAAGCTTTCAA	Ahringer library
28	*apb-1*	Y71H2B.10	AAAATTCGGCTCAAACATCTGTA	GATTTTACAGGAACTCCAATCCC	Ahringer library
29	*apm-1*	F55A12.7	ATCGACATGTCGATTTCCGGTCTCTTC	ACGGAGTACGGTTCCTTGAGCATTTG	Conctructed
30	*apg-1*	Y105E8A.9	CTGTCGAGCTAGCCATTGAGAAGATC	AAGCGAATTAGTGACGAGCATATGAAC	Conctructed
31	*smap-1*	W09D10.1	AGACTGCAAGGCTTCCTGTTGGACATG	CCGAGACAAATGATCCGAAATCGTCG	Conctructed
32	*apt-9*	W04G3.4	CCATTGACTACTGGGTTTGTAAGTGACAC	TCAAACACCGATGACCTGACAGGTG	Conctructed
33	*chc-1*	T20G5.1	GTGAAGCAAATCCCACTGGT	TTATTGATGCGGGGAGAAAG	Ahringer library
34	*sec-8*	Y106G6H.7	ACATGCGGAATTTGGAGTTC	CTGCTGGTGTCATTGGAGAA	Ahringer library
35	*exoc-7*	C43E11.8	TGCGATGATTTCTGTGCTTC	AAATTCCAAAAATTGCGTGG	Ahringer library
36	*sec-5*	T23G7.4	TCAGGAGAAGCCGTTGAGTT	ATCCGCAGTCTGGGTAGAT	Ahringer library
37	*sec-3*	F52E4.7	CACAAAGTTTGCAAAGCGAA	TCGCTAAAGGACCCTCTGAA	Ahringer library
38	*exoc-8*	Y105E8B.e	TCCCATGATCTTCCATTTTCTTA	CTAAATTTCAAATGGGGAAAAGG	Ahringer library
39	*sec-10*	C33H5.9	CGACATCTTCAGCGACAAAA	GAAAATGCGATTCGAGGAAG	Ahringer library
40	*sec-15*	C28G1.3	CAATCCGATGACATCACGAG	CGGTCAACTCGGAAGAAGAG	Ahringer library
41	*sec-6*	F09E5.5	AGCAAGTAGCAGCTCTACTACAACG	GCTTCGAGCTTCCATGTAGAATTTATCGA	Conctructed
42	*rab-11.1*	F53G12.1	ATGGGCTCTCGTGACGATGAATACG	CGAAGGAATGATTGTACCACTGCCACC	Conctructed
43	*rab-11.2*	W04G5.2	ATGGGCAACGAATACTACTACTTGTTCAAG	GCTTTCCATAAATAAATTTTTCGACAGATT	Ahringer library
44	*rab-8*	D1037.4	GAGAGCGGACACAGAGAAATAGA	CTCACTCACTCCAATAAAATGGC	Ahringer library
45		F54C9.11	CCGCAAGTTTACAACGTGAA	TGTGCTTCTTCTTCTCCGGT	Ahringer library
46	*rab-35*	Y47D3A.25	cDNA		RCE library
47	*rab-10*	T23H2.5	cDNA		RCE library
48	*amph-1*	F58G6.1	GAGCATCACTCGATGCAAGA	AGCAACGAATCGTACCAAGG	Ahringer library
49	*sdpn-1*	F45E1.7	CGAATCAGGTTCTGTGAGCA	GATTGGGTGTGTCTGGGTTC	Ahringer library
50	*alx-1*	R10E12.1	ATCGACTAATGAAGTGGATCTTGTGAAGCC	GCTGCTGCTAAGAAGGATAACGATTTTATA	Conctructed
51	*rme-1*	W06H8.1	AGAAGAACAAGGAAGTTTTGGAGACGG	TGCTAAAGTCCTGTGTTTGAAGCTTC	Conctructed
52	*snap-29*	K02D10.5	cDNA		RCE library
53	*ehbp-1*	F25B3.1	CAAATGATTCCAGAGCAGCAGGTAT	AATCTTTGAAGGATTTGACGAGGGA	Ahringer library
54	*arf-6*	Y116A8C.12	cDNA		RCE library
55	*sid-3/kin-25*	B0302.1	AGCGTAATCAGTGGTGGGTCGAACA	CTGTGCCATCAACTGCAAGAGACAA	Ahringer library
56	*sac-1/sacm-1L*	F30a10.6	TTCCCTTTTCAGCTCTCCAAAATGG	CAACCGATGCCTACACTGAAACCAA	Ahringer library
57	*ptrn-1/pqn-34*	F35B3.5	CAACACCACATTGCAACACATTTAA	GAGCTCTCAGCTAGGTCAACCGGAA	Ahringer library
58	*rtkn-1*	C07B5.4	CAGCACGAACTGATTACGGACTTGA	ATCTGCTTCCGGTTCCAGTCATCAT	Ahringer library
59	*syx-17*	VF39H2L.1	CATTAGTGCGCATGTATGAAAAA	AAAGAACTCTCAATCACTCGTGG	Ahringer library
60	*snt-4*	T23H2.2	GGCTCTGGCAAATCCAATTA	GACGAAAGGAACTGCGAGTC	Ahringer library
61	*snt-2*	F42G9.7	CTTGCTCATCTTGTCATAATCCC	GTTCTGCCAGAGAGAAAACAGAA	Ahringer library
62	*rab-27*	Y87G2A.4	cDNA		RCE library
63	*rab-3*	C18A3.6	AGCTCTGGGAATTGTTTGGA	CCAATCTTCGAAGGGTTCAA	Ahringer library
64	*unc-64/syx-1*	F56A8.7	cDNA		RCE library
65	*syn-1/syx-3*	F35C8.4	AAGCAACAGTTAACACTATACATG C	TTCAGATACTCTAGATTCCAATCA ATTAC	Conctructed
66	*syx-6*	C15C7.1	ATGAGCAACTACCGATACTCAAAACTC	TCAGACCAGAAGGACAAAGACG	Conctructed
67	*syn-13/syx-7*	F36F2.4	ATGAGTATATCGGAGTGCTCAACCG	ACAGAGACACTCCTATTACTTAGCCAGG	Conctructed
68	*ric-4/snap-25*	Y22F5A.3	TGACAACTCATTCCACCTGC	GACGGAATTTGCAAAAGCA	Ahringer library
69	*aex-4*	T14G12.2	cDNA		RCE library
70	*snb-7*	ZK795.4	AAGATCAAAGCCTGTCAAGACAGTTTC	AATGGAGTGGTTCACAAAGCAGTGC	Conctructed
71	*snb-5*	C30A5.5	TTACTGCATGAATCATCAACCAC	CACAAAGCGTAATGCCAATAAAT	Ahringer library
72	*snb-6*	T14D7.3	AACTAACTGTGACGTGGGAAAAA	ACCGATAATTGGGAAAAGTTTGT	Ahringer library
73	*vamp-7*	Y69A2AR.6	AGTGAGCCTAAAATCATCGATTCTTGC	TCCATAATTCGCTCGACATTGTCC	Conctructed
74	*vamp-8*	B0513.9	ACGACAGCAACAAGCCCAGCGACAATG	TTCCAATGACTCCAGTCCAATGAAG	Conctructed
75	*gos-28/gosr-1*	F08F8.8	TGCGTTGTATGAAGGGACAA	CTCGGCATCTTGGGCTAAT	Ahringer library
76	*ykt-6*	B0361.10	ATGTGAAGCTGTTTAAATCGGAATGTGATC	TTAGTTTGCTTCTCGGTATTTCTTGGAAAT	Ahringer library
77	*snt-6*	C08G5.4	ACGAGCAAAATACGTAATCCAAA	AAATTGACTTTTGTGCTCCGTTA	Ahringer library
78	*cup-5/muc-1*	R13A5.1	TGGAATGACGACAGAGATGC	TAGATGGGCTTTGATGAGGG	Ahringer library
79	*unc-116*	R05D3.7	CTGCTTGTGAACCTTCGTCA	GAAGAATGGAAACGGCGATA	Ahringer library

Supporting_Files S1_Mutated_sequence_of_*rme-1*

> *rme-1(hq635)*_5bp-deletion

…AATCGATCGCGGATATGATTTCACTGGAGTTTTAGAGTGGTTCGCTGAACGTGTCGATCGTATCATTCTTCT
TTTCGATGCTCACAAGCTTGACATTTCCGATGAGTTCAAGAGATGTATTGAAGCATTGGCTGGAAACGAGGA
TAAAATACGAATTGTTCTCAACAAGTCAGACATGGTTGATCATCAACAGTTGATGAGAGTCTACGGAGCTCT
GATGTGGTCACTGGGAAAAGTATTCAAAACTCCAGAAGTTTCACGTGTCTATTTGGGATCATTCTGGGATCAT
CCACTTCACTACGATCTCAATCGTCGACTTTTCCAAGACGAGCAGCACGACCTGTTCCAAGATCTTCAAGCAT
TGCCACG~~TAACG~~CTGCTCTCCGAAAGTTGAACGATCTGATCAAGAGAGCTCGTCTCGCCAAGGTTCACGCAT
ACATTATTGCTGAGCTTCGTAAGCAAATGCCATCGATGATCGGAAAGGATAAGAAGAAGAAGGATTTGATCC
AAAATCTTGACAAGATCTACGAGCAACTCCAGAGAGAACACAATATATCTCCAGGAGACTTCCCAGATGTGA
ACAAG…

> *rme-1(hq636)*_9bp-insertion+44bp-deletion

…AATCGATCGCGGATATGATTTCACTGGAGTTTTAGAGTGGTTCGCTGAACGTGTCGATCGTATCATTCTTCT
TTTCGATGCTCACAAGCTTGACATTTCCGATGAGTTCAAGAGATGTATTGAAGCATTGGCTGGAAACGAGGA
TAAAATACGAATTGTTCTCAACAAGTCAGACATGGTTGATCATCAACAGTTGATGAGAGTCTACGGAGCTCT
GATGTGGTCACTGGGAAAAGTATTCAAAACTCCAGAAGTTTCACGTGTCTATTTGGGATCATTCTGGGATCAT
CCACTTCACTACGATCTCAATCGTCGACTTTTCCAAGCATACGGAG~~ACGAGCAGCACGACCTGTTCCAAGAT~~
~~CTTCAAGCATTGCCACGT~~AACGCTGCTCTCCGAAAGTTGAACGATCTGATCAAGAGAGCTCGTCTCGCCAAG
GTTCACGCATACATTATTGCTGAGCTTCGTAAGCAAATGCCATCGATGATCGGAAAGGATAAGAAGAAGAAG
GATTTGATCCAAAATCTTGACAAGATCTACGAGCAACTCCAGAGAGAACACAATATATCTCCAGGAGACTTC
CCAGATGTGAACAAG…

> *rme-1(hq637)*_23bp-deletion

…AATCGATCGCGGATATGATTTCACTGGAGTTTTAGAGTGGTTCGCTGAACGTGTCGATCGTATCATTCTTCT
TTTCGATGCTCACAAGCTTGACATTTCCGATGAGTTCAAGAGATGTATTGAAGCATTGGCTGGAAACGAGGA
TAAAATACGAATTGTTCTCAACAAGTCAGACATGGTTGATCATCAACAGTTGATGAGAGTCTACGGAGCTCT
GATGTGGTCACTGGGAAAAGTATTCAAAACTCCAGAAGTTTCACGTGTCTATTTGGGATCATTCTGGGATCAT
CCACTTCACTACGATCTCAATCGTCGACTT~~TTCCAAGACGAGCAGCACGACCT~~GTTCCAAGATCTTCAAGCAT
TGCCACGTAACGCTGCTCTCCGAAAGTTGAACGATCTGATCAAGAGAGCTCGTCTCGCCAAGGTTCACGCAT
ACATTATTGCTGAGCTTCGTAAGCAAATGCCATCGATGATCGGAAAGGATAAGAAGAAGAAGGATTTGATCC
AAAATCTTGACAAGATCTACGAGCAACTCCAGAGAGAACACAATATATCTCCAGGAGACTTCCCAGATGTGA
ACAAG…

> *rme-1(hq638)*_7bp-deletion

…AATCGATCGCGGATATGATTTCACTGGAGTTTTAGAGTGGTTCGCTGAACGTGTCGATCGTATCATTCTTCT
TTTCGATGCTCACAAGCTTGACATTTCCGATGAGTTCAAGAGATGTATTGAAGCATTGGCTGGAAACGAGGA
TAAAATACGAATTGTTCTCAACAAGTCAGACATGGTTGATCATCAACAGTTGATGAGAGTCTACGGAGCTCT
GATGTGGTCACTGGGAAAAGTATTCAAAACTCCAGAAGTTTCACGTGTCTATTTGGGATCATTCTGGGATCAT
CCACTTCACTACGATCTCAATCGTCGACTTTTCCAAGACGAGCAGCACGACCTGTTCCAAGATCTTCAAGCAT
TGCC~~ACGTAAC~~GCTGCTCTCCGAAAGTTGAACGATCTGATCAAGAGAGCTCGTCTCGCCAAGGTTCACGCAT
ACATTATTGCTGAGCTTCGTAAGCAAATGCCATCGATGATCGGAAAGGATAAGAAGAAGAAGGATTTGATCC
AAAATCTTGACAAGATCTACGAGCAACTCCAGAGAGAACACAATATATCTCCAGGAGACTTCCCAGATGTGA
ACAAG…
